# Zinc finger knuckle genes are associated with tolerance to drought and dehydration in chickpea (*Cicer arietinum* L.)

**DOI:** 10.3389/fpls.2024.1354413

**Published:** 2024-05-03

**Authors:** Gulmira Khassanova, Irina Oshergina, Evgeniy Ten, Satyvaldy Jatayev, Nursaule Zhanbyrshina, Ademi Gabdola, Narendra K. Gupta, Carly Schramm, Antonio Pupulin, Lauren Philp-Dutton, Peter Anderson, Crystal Sweetman, Colin L.D. Jenkins, Kathleen L. Soole, Yuri Shavrukov

**Affiliations:** ^1^ Faculty of Agronomy, S.Seifullin Kazakh AgroTechnical Research University, Astana, Kazakhstan; ^2^ Department of Crop Breeding, A.I.Barayev Research and Production Centre of Grain Farming, Shortandy, Kazakhstan; ^3^ Department of Plant Physiology, Sri Karan Narendra (SNK) Agricultural University, Jobster, Rajastan, India; ^4^ College of Science and Engineering (Biological Sciences), Flinders University, Adelaide, SA, Australia

**Keywords:** CCHC domain, chickpea, DArT analysis, drought and dehydration, gene expression, seed yield, SNP, zinc finger knuckle gene

## Abstract

Chickpea (*Cicer arietinum* L.) is a very important food legume and needs improved drought tolerance for higher seed production in dry environments. The aim of this study was to determine diversity and genetic polymorphism in zinc finger knuckle genes with *CCHC* domains and their functional analysis for practical improvement of chickpea breeding. Two *CaZF-CCHC* genes, *Ca04468* and *Ca07571*, were identified as potentially important candidates associated with plant responses to drought and dehydration. To study these genes, various methods were used including Sanger sequencing, DArT (Diversity array technology) and molecular markers for plant genotyping, gene expression analysis using RT-qPCR, and associations with seed-related traits in chickpea plants grown in field trials. These genes were studied for genetic polymorphism among a set of chickpea accessions, and one SNP was selected for further study from four identified SNPs between the promoter regions of each of the two genes. Molecular markers were developed for the SNP and verified using the ASQ and CAPS methods. Genotyping of parents and selected breeding lines from two hybrid populations, and SNP positions on chromosomes with haplotype identification, were confirmed using DArT microarray analysis. Differential expression profiles were identified in the parents and the hybrid populations under gradual drought and rapid dehydration. The SNP-based genotypes were differentially associated with seed weight per plant but not with 100 seed weight. The two developed and verified SNP molecular markers for both genes, *Ca04468* and *Ca07571*, respectively, could be used for marker-assisted selection in novel chickpea cultivars with improved tolerance to drought and dehydration.

## Introduction

Chickpea (*Cicer arietinum* L.) is an important food legume in many countries due to high levels of seed proteins and high nutrient value (Didinger and Thompson, 2022; Koul et al., 2022). The chickpea plant shows a degree of tolerance to several abiotic stresses, including drought, dehydration, heat, and salinity, making it a versatile crop plant (Arriagada et al., 2022; Asati et al., 2022; Karalija et al., 2022). However, there is room for development of new chickpea cultivars with improved growth in harsh environments, higher yield, and better seed quality (Eker et al., 2022a, Eker et al., 2022b). To achieve this goal most efficiently, the identification of the most important genes requires molecular breeding tools (Jain et al., 2023). The genomic and genetic study of chickpea is very well established (Varshney et al., 2021; Ali et al., 2022; Bohra et al., 2022) with fully sequenced genomes of cv. Frontier (Kabuli ecotype) and the accession ICC-4958 (Desi ecotype) now publicly available to researchers in databases Legume Information System (LIS, https://www.legumeinfo.org) and Chickpea Portal (http://www.cicer.info/databases.php). Within these databases, genes with zinc finger motifs can be identified.

Gene families with zinc finger (ZF) sequences form a large group and are widely distributed in all living organisms. Within these, zinc finger knuckles with CCHC (or C_2_HC) domain are less well characterized compared to other ZF families. Initially, ZF-CCHC was reported in retroviruses like HIV, where nucleocapsid proteins containing CCHC domains play important roles in the viral lifecycle (Armas and Calcaterra, 2012). The CCHC domain is highly conserved with a simple consensus sequence as follows: Cys–X_2_–Cys–X_4_–His–X_4_–Cys, where X can be any amino acid. Any change in the critical zinc-binding amino acids, i.e., any of the three Cys or His, results in a protein that is defective for RNA binding and viral packaging. ZF-CCHC proteins are found in microorganisms, yeast, plants, animals, and humans and can contain as few as 1 CCHC domain or as many as 11. These proteins play essential functions in the metabolism of nucleic acids and mediation of protein–protein interactions (Liew et al., 2000). CCHC domains, when located in the N-termini of ZF proteins, are often involved in homodimerization of transcription factors resulting either in activation or repression of various biological processes in animals based on the example of a mouse model (Grabarczyk et al., 2018).

In plants, ZF-CCHC proteins were first described as RNA-binding proteins, and they have since been found to play important roles in plant growth, development, and stress responses (Reviewed in: Lee and Kang, 2016). Recently, in a comprehensive study of bread wheat (*Triticum aestivum* L.), 50 TaZF-CCHC proteins and their corresponding genes were identified and found to be distributed in nine clusters in a molecular dendrogram (Sun et al., 2022). The authors studied 38 out of the 50 genes, and 32 of them were found to be either up- or downregulated in response to drought, heat stress, or both. Some, but not all, of the genes had similar expression patterns (Sun et al., 2022).

Individual *ZF-CCHC* genes have been functionally described for various plant species. Arginine/serine (RS)-rich splicing factors with a CCHC domain, AdRSZ21 and AtRSZp22, were confirmed to be related to plant defense and cell death via the hypersensitive response to pathogen infection found in *Arachis diogoi* (Kumar and Kirti, 2012) and in *Arabidopsis thaliana* with homologs in mammals and humans (Lopato et al., 1999). Other glycine-rich RNA-binding proteins (RZ) play an important role in plant growth regulation and improving plant resistance to bacterial infections in bread wheat (Xu et al., 2015) and to ascochyta blight in chickpea (Iruela et al., 2009). Similar RZ proteins were identified in the genus *Eucalyptus*, but their function remains unclear (Bocca et al., 2005).

The specific ZF-CCHC genes, *AIR1* and *AIR2*, encode arginine methyltransferase-interacting proteins with five CCHC domains, and they were described as important RNA-binding proteins in yeast acting as nuclear cofactors for RNA degradation via exosomes (Fasken et al., 2011; Lange and Gagliardi, 2022). Similarly, gene *GIS2*, glucose inhibition of gluconeogenic growth-suppressor 2, was first described in yeast as encoding a cytoplasmic ZF protein with seven “retroviral”-type CCHC domains and predicted to interact with DNA and possibly RNA (Scherrer et al., 2011). The AIR1, AIR2, and GIS2 proteins were not described in *A. thaliana* (L.) Heynh (Aceituno-Valenzuela et al., 2020), but similar proteins were found in bread wheat and rice (Sun et al., 2022).

Much better known are glycine-rich proteins (GRP) containing both a CCHC and a cold shock domain (CSD). This group of cold shock proteins (CSP) or cold shock domain proteins (CSDP) is very wide and intensively studied in many plant species in response to cold and freezing but not in other abiotic stresses (Sasaki and Imai, 2012). For example, in *A. thaliana*, GRP2 proteins contain both zinc finger CCHC and a cold shock domain (Kingsley and Palis, 1994), and contribute to the enhancement of freezing tolerance (Kwak et al., 2011). The expression levels of many of these genes in *A. thaliana* were upregulated by cold stress but not by drought or salinity (Kim et al., 2007, Kim et al., 2010, Kim et al., 2013).

In other plant species, for example, in saltwater cress (*Eutrema salsugineum* Pall.), CSDP proteins with a varying number of CCHC-domains in the C-terminus were found to be similar to CSDP1 and CSDP2 in *A. thaliana* (Taranov et al., 2018). In bread wheat, the gene *WCSP1* (wheat cold shock protein 1) was reported to be gradually upregulated after cold treatment but otherwise not affected by other abiotic stresses, like drought, salinity, or heat stress (Karlson et al., 2002). In rice (*Oryza sativa* L.), two genes encoding cold shock domain proteins, *OsCSP1* and *OsCSP2*, also showed increased expression, but only after a very short exposure of 0.5–1 h of cold with a subsequent return back to the initial level (Chaikam and Karlson, 2008). In cabbage (*Brassica oleracea* L.), genetic polymorphism in the *BoCSDP5* gene did not affect the gene expression levels, but other ZF-proteins were produced, with varying numbers of CCHC domains, in plants with different alleles of the *BoCSDP5* gene (Song et al., 2020).

Additionally, CSDP1 and CSDP2 proteins with seven and two CCHC domains, respectively, had an important effect on seed germination in *Arabidopsis*. The overexpression of the corresponding genes rescued a mutant for glycine-rich RNA-binding protein from cold damage (Park et al., 2010). Significantly earlier-flowering transgenic *Arabidopsi*s plants were reported after silencing of the similar gene *AtGRP2* encoding a glycine-rich protein with both CSD and CCHC domains (Fusaro et al., 2007). In poplar (*Populus* × *xiaohei* T.S. Hwang et Liang), “Zn-finger (CCHC)” genes were identified in RNA-seq transcriptome profiling of seed germination, but exact identification of the genes and encoded ZF-CCHC proteins were not provided (Qu et al., 2019).

Proteins containing a group of three amino acids: glycine–arginine–phenylalanine, designated by “GRF,” encoding GRF-type ZF proteins are widely distributed throughout eukaryotes, but in plants, their structure is very different to yeast and humans sharing similarity only in the main components (Aceituno-Valenzuela et al., 2020). In *Xenopus*, protein ZF-CCHC4 with a GRF domain binding a Zn^2+^ ion plays a central structural role in coordination with a partner CCHC domain (Wallace et al., 2017). GRF-ZF proteins are involved in DNA damage response, transcriptional regulation, and RNA metabolism both in animals and plants (Aceituno-Valenzuela et al., 2020; Sun et al., 2022).

Attention has to be paid to the zinc finger CCHC-type protein in the model legume species *Medicago truncatula* Gaertn (Radkova et al., 2019, Radkova et al., 2021). This MtZF-CCHC protein (ABE91952) was unique and related to flower morphology and seed size in transgenic *M. truncatula* and *A. thaliana* lines. Overexpression of the *MtZF-CCHC* transgene caused taller plants with larger seed size, whereas the exact opposite effect was demonstrated in knockdown expression in RNAi lines, with strongly reduced seed size accompanied by shorter stem length and internodes (Radkova et al., 2019, Radkova et al., 2021).

*ZF-CCHC* genes have been reported to play important roles in plant responses to various abiotic stresses. For example, in rice, the nuclear-localized protein encoded by the *OsZFP6* gene was upregulated in response to abiotic factors like salinity, alkalinity, and H_2_O_2_ oxidative stress. These results were confirmed with transgenic yeast and *Arabidopsis* plants with *OsZFP6* overexpression, where tolerance to these abiotic stresses was significantly increased (Guan et al., 2014). The *OsZFP6* gene (LOC_Os02g34760 = LOC4329640) belongs to the retrotransposon GAG polyprotein (Sun et al., 2022). Additionally, two genes, *OsCSP1* and *OsCSP2*, were upregulated in roots and downregulated in shoots of rice seedlings exposed to dehydration (Chaikam and Karlson, 2008). Ecotypes of the reed, *Phragmites communis* Trin, a hydrophytic species can adapt well to harsh drought conditions, and proteomics analysis indicated that at least two groups of ZF-CCHC proteins are present during plant adaptation (Li et al., 2021). They include a serine-/arginine-rich protein, similar to wheat described above (Sun et al., 2022), and chloroplastic protein DEAD-box ATP-dependent RNA helicase with homology to rice, Os03t0827700 (Sun et al., 2022), *Arabidopsis*, and maize (*Zea mays* L.) (Aceituno et al., 2020).

In plant research, assessment of genetic diversity is mostly based on single-nucleotide polymorphism (SNP) analysis, with an enormous number of publications completed to date (Reviewed in: Huq et al., 2016; Morgil et al., 2020), and more specifically in chickpea (Basu et al., 2018). There are many methods available for plant genotyping based on SNP analysis (Schramm et al., 2019), including next-generation sequencing (Shavrukov et al., 2014). These include more traditional and manual techniques that are slow and have low throughput to more expensive methods like cleaved amplified polymorphic sequences (CAPS), which requires the following three steps: PCR-based amplification of a specific genetic fragment, digestion with a restriction enzyme where the recognition site specifically targets the SNP, and separation of digested fragments on gels (Reviewed in: Shavrukov, 2016). In general, it is much faster to work with medium throughput methods, for example, based on fluorescence (Förster) resonance energy transfer (FRET). Recently, allele-specific qPCR (ASQ) methods for plant genotyping have been developed that are also suitable for chickpea (Kalendar et al., 2022; Amangeldiyeva et al., 2023). Microarray technology employing thousands of simultaneous reactions on a microchip represents the next generation of plant genotyping (Varshney et al., 2012), and diversity array technology (DArT) is also very powerful and popular for the identification of SNP and haplotypes (a group of closely located SNPs) in genetic regions of entire genomes (James et al., 2008; Deres and Feyissa, 2022), including chickpea (Roorkiwal et al., 2014; Thudi et al., 2014).

However, for functional analysis, it is important to show how gene expression is changed in response to stress treatment or during plant development. RT-qPCR or RNA-seq technology thus becomes a reasonable requirement for gene studies in plants (Upton et al., 2023), including chickpea (Nguyen et al., 2015; Borhani et al., 2020). Based on genotyping and gene expression analysis, the final step for “proof of a hypothesis” is to show how selected genotypes are more tolerant to stresses and how seed yield and quality are improved. This is a classical combination of marker-assisted selection (MAS) with proven functional analysis of the candidate genes (Boopathi, 2020), and such a strategy is applicable for crop improvement in general (Ben-Ari and Lavi, 2012) and for chickpea specifically (Basu et al., 2018; Chahande et al., 2021).

The current study represents the first step in determining the diversity and genetic polymorphism in *CaZF-CCHC* and their functional analysis for practical improvement of chickpea breeding. The following strategies were used: (1) molecular–phylogenetic analysis of CaZF-CCHC proteins in chickpea and other legume species, (2) SNP analysis in two *CaZF-CCHC* genes (*Ca04468* and *Ca07571*) selected as potential candidates for improved plant response to drought, (3) SNP genotyping using ASQ and CAPS markers in parents and hybrid breeding lines of chickpea, (4) 6K DArT microarray analysis for haplotypes of genetic regions in the selected hybrid breeding lines, (5) RT-qPCR expression analysis of *Ca04468* and *Ca07571* genes in parents and hybrid breeding lines under drought and dehydration, and (6) evaluation of yield-related traits in chickpea genotypes in field trials in Kazakhstan under mild and strong drought conditions.

## Materials and methods

### Identification of zinc finger genes with CCHC domains, *Ca04468* and *Ca07571*, and encoded proteins

A candidate gene from the family *CaZF-CCHC* in chickpea with a potential role in tolerance to abiotic stresses was identified in the NCBI database for SNP in *C. arietinum* L. (https://www.ncbi.nlm.nih.gov/snp). The gene family with full-length nucleotide sequences and corresponding polypeptide sequence was retrieved from the same database and used for both BLASTN and BLASTP in databases NCBI and GenomeNet, Kyoto University, Japan (https://www.genome.jp/tools/blast). After the identified *CaZF-CCHC* gene family was studied, based on differences in SNP databases, two genes, *Ca04468* and *Ca07571*, with a specific combination of Zn-finger domains, were selected for further analysis as the most suitable candidates for plant response to drought and dehydration. All sequences of genes and encoded proteins in chickpea and other legume species were identified and downloaded from GenomeNet and NCBI databases. Chromosome locations, positions on the physical map, and gene identification for chickpea was found in the Legume Information System database (LIS) using BLAST, cv. Frontier v1.0 assembly (https://www.legumeinfo.org/taxa/cicer).

The molecular–phylogenetic dendrogram was constructed using CLUSTALW Multiple Sequence Alignment at GenomeNet Database Resources (https://www.genome.jp/tools/blast) with the ETE3 FastTree program. The result file was converted into a “nex” file for further use in SplitsTree4 version 4.14.4 from algorithms in the bioinformatics website at the University of Tübingen, Germany (https://uni-tuebingen.de).

### Plant material and hybridization

Initially, six chickpea germplasm accessions were studied, including three Kabuli-type cultivars, Kamila, Krasnokutsky-123, and Looch, and three Desi-type accessions, ICC-1083, ICC-10945, and ICC-12654. Additionally, accession ICC-4958 was used as a reference genotype. Seeds of the Kabuli-type cultivars were obtained from the collection of S.Seifullin Kazakh AgroTechnical Research University, Astana, Kazakhstan, and included locally adapted genotypes originating from both Russia and Kazakhstan. The remaining chickpea accessions were obtained from the ICRISAT Reference set collection, India, and distributed for research purposes in Australia.

Selected chickpea accessions were crossed using manual emasculation and controlled pollination. The true hybrid F_1_ plants were confirmed by flower color, with maternal and paternal parents bearing white and colored flowers, as well as PCR analysis of the marker KATU-C22 developed earlier (Khassanova et al., 2019). Each F_2_ plant was used as the progenitor of breeding line families, and F_6_ breeding lines were used in the analysis. For the purpose of this study, two hybrid populations were studied as follows: (1) ♀ Krasnokutsky-123 × ♂ ICC-12654 and (2) ♀ ICC-10945 and ♂ Looch. Cultivars Krasnokutsky-123 (with dark seeds) and Looch (with light seeds), Kabuli-type, originated from Russia and Kazakhstan, respectively. Chickpea ICRISAT accessions, ICC-12654 and ICC-10945, both with dark seeds, Desi-type, originated from Ethiopia and India, respectively. In hybrid populations 1 and 2, originally 12 F_6_ breeding lines were developed in each population with subsequent selection of six and seven, respectively, for DArT analysis, and three and four for qPCR and field trial testing, respectively.

### Drought and dehydration experiments

Seeds were sown in 6-L plastic pots filled with BioGro, Australia, potting media with the addition of “NoduleN” chickpea legume inoculant, New Edge Microbial, Australia. For each genotype, four plants per pot and two pots per treatment were grown in a greenhouse with 26°C/18°C day/night, with natural light and, on cloudy days, supplemented with LED with a photon flux density of 800 µmol m^−2^·s^−1^. The relative humidity was controlled at 40%, and pots were watered with tap water twice per week. Placement of plants in the greenhouse was fully randomized.

For slowly developed drought, plants were grown for 1 month in well-watered conditions. Four time-points were designated for sampling starting from “day 0,” before the start of drought treatment, and 5, 7, and 9 days after watering was withdrawn. Leaf wilting symptoms in stress-treated plants were observed gradually. Watering was continued unchanged for control plants. Leaf samples were collected from three randomly selected plants (three biological replicates) for each time-point, genotype, and treatment, then frozen in liquid nitrogen and stored at −80°C for further analyses.

For rapid dehydration of detached leaves, a separate set of 1-month-old well-watered plants was used. For each genotype, leaf samples were split into four batches, designated as 0, 2, 4, and 6 h, with three biological replicates in each batch. The first group of samples (0 h) was frozen immediately in liquid nitrogen, whereas other leaf samples were exposed to dehydration on paper towel on the lab bench at room temperature (22°C). Consequently, after 2, 4, and 6 h of exposure to dehydration, leaf samples were frozen in liquid nitrogen and stored for further analyses.

### DNA extraction, sequencing, and SNP identification

DNA was extracted as described earlier (Weining and Langridge, 1991; Khassanova et al., 2019) with the following adjustments. Leaf samples were collected in 10-ml tubes from individual plants and frozen in liquid nitrogen. They were ground with two 8-mm stainless ball bearings using a Vortex mixer. One microliter of DNA was checked on a 1% agarose gel to assess quality, and concentration was measured by Nano-Drop (Thermo Fisher Scientific, USA).

To identify SNPs in the identified gene fragments, they were compared with reference genotypes of cv. Frontier and accession ICC-4958, then primers were designed in coding regions and promoters (**Supplementary Material S1**) covering approximately 1 kb of amplification fragments. PCR was performed in 60-µl-volume reactions containing 6 µl of template DNA adjusted to 50 ng/ml and with the following components in their final concentrations as listed: 1× PCR buffer, 2 mM of MgCl_2_, 0.2 mM each of dNTPs, 0.25 mM of each primer, and 4.0 U of GoTaq Flexi DNA polymerase (Promega, USA) in each reaction. PCR was conducted on a Thermal iCycler (Bio-Rad, USA) using a program with the following steps: initial denaturation, 94°C, 2 min; 35 cycles of 94°C for 15 s, 55°C for 15 s, 72°C for 1 min, and a final extension of 72°C for 3 min. Single bands of the expected size were confirmed after visualization of 5 µl of the PCR product in 1% agarose gel. The remaining PCR reaction volume (55 µl) was purified using a FavorPrep PCR Purification kit (Favorgene Biotec Corp., Taiwan) following the manufacturer’s protocol. The concentrations of purified PCR products were measured using NanoDrop (Thermo Fisher Scientific, USA) and later used as a template for Sanger sequencing at the Australian Genome Research Facility (AGRF), Adelaide, Australia. SNPs were visualized using the Chromas computer software program version 2.0 with manual comparison and SNP identification.

### SNP genotyping using ASQ and CAPS methods

The ASQ method was used for plant genotyping following the protocol described recently (Amangeldiyeva et al., 2023). In brief, option A with a short 4-bp tag was used, and two allele-specific forward primers and one reverse primer were developed targeting two SNPs, *Ca04468*-SNP1 and *Ca07571*-SNP4. The sequence of the primers and three universal probes with attached FAM, HEX, and quencher Dabcyl are presented in **Supplementary Material S1**. Primers and universal probes were ordered from Sigma-Merck (Australia). Master-mix preparation was the same as described by Amangeldiyeva et al. (2023). Each reaction had a 10-µl cocktail in total and was loaded in a 96-well BioRad microplate sealed with clear tape prior to amplification in a CFX96 Real-Time PCR Detection System (Bio-Rad, USA) with automatically recorded fluorescence using the described protocol (Amangeldiyeva et al., 2023). Amplification of FAM and HEX was checked and controlled, whereas genotyping results were determined in a post-run step automatically using the software CFX Manager accompanying the qPCR instrument.

The CAPS method was used for verification of *Ca07571-*SNP4. Primers were re-designed to make the amplicon shorter to avoid multiple cutting. Sequences of the primers are present in **Supplementary Material S1**. The PCR reaction mix was as described above for sequencing but reduced 2× proportionally for all components with a total PCR volume of 30 µl. The PCR amplification protocol was also adjusted for shorter steps but maintained the same temperatures as follows: initial denaturation, 94°C, 2 min; 35 cycles of 94°C for 10 s, 55°C for 10 s, 72°C for 25 s, and final extension, 72°C for 1 min. After amplification, the entire 30-µl reaction was subjected to digestion with 4 µl (20 U of enzyme activity) of *Mnl*I (NEBiolab, England), 4 µl of supplied 10× CutSmart buffer, and 2 µl of sterile water making 40 µl of total digestion mix volume. After 2 h of digestion in an incubator at 37°C, digested PCR products were separated by running in 12% polyacrylamide gel, visualized with ethidium bromide using a GelDoc imaging system (BioRad, USA). In both ASQ and CASP methods, the sources of potential error could be related to the accuracy of instruments, “human error,” and reproducibility of the received results. All experiments were carried out with three biological replicates (plants) and two technical replicates (repeated runs) for each studied sample, so that all potential errors are eliminated in the preliminary steps.

### DArT microarray analysis

DNA extracted from leaves of individual plants as described above was adjusted to 100 ng/µl and aliquoted into 50-µl volumes and submitted to Diversity Array Technology Co., Canberra, Australia (https://www.diversityarrays.com) for genotyping using chickpea DArTseq (1.0) with 6K DArT clones. Results were presented in two major files with the Silico-DArT and SNP map used for further analysis.

### RNA extraction and RT-qPCR analysis of gene expression

Leaf samples were collected from individual plants, frozen, and ground as described for DNA extractions. TRIzol-like reagent was used for RNA extraction following the protocol developed earlier (Shavrukov et al., 2013), then cDNA synthesis and RT-qPCR analysis as described previously (Sweetman et al., 2020). Briefly, after 1 µl of DNase treatment (NEBiolab, England) and reverse transcription with 2 μg of RNA using the Protoscript Reverse Transcriptase kit (NEBiolab, England), cDNA samples were diluted with sterile water (1:10), resulting in a DNA quantity of around 10 ng of cDNA, and used for RT-qPCR analysis. KAPA SYBR Fast Universal Mix (KAPA Biosystems, USA), was used in 10-µl total volume containing 0.5 µM primers and 3 µl of cDNA, and run in a CFX96 Real-Time qPCR system (BioRad, USA). Thermal cycling conditions involved an initial melt at 95°C for 3 min, followed by 40 cycles of 95°C, 5 s, and 60°C, 20 s, with post-PCR melt curve from 60°C to 95°C increasing by 0.5°C increments every 5 s. Expression levels of target genes were normalized relative to the geometric average of two reference gene transcript levels (Bustin et al., 2009): *CaELF1α*, elongation factor 1-alpha (AJ004960) and *CaHsp90*, and heat shock protein 90 (GR406804) (Garg et al., 2010). Sequences of all gene-specific and reference primers are present in **Supplementary Material S1**. At least three biological replicates (individual plants) and two technical repeats were used for each genotype and treatment.

### Field experiments and seed-related traits analysis

For field experiments, the same three F_6_ breeding lines and parents were used from hybrid population 1 (♀ Krasnokutsky-123 × ♂ ICC-12654) and four F_6_ breeding lines and parents from hybrid population 2 (♀ ICC-10945 × ♂ Looch). Field experiments were carried out in 2021 and 2022 in the Akmola region (Kazakhstan) in a research field of S.Seifullin Kazakh AgroTechnical Research University. Volumetric water content (VWC) in the soil was measured using a portable Moisture meter (Model CS616, Campbell Scientific, Australia). At the stage of fully developed plants (45 days since seed sowing), VWC value was decreased from 80% field capacity (sowing time) to 48% (mild drought) in 2021 and to 35% (strong drought) in 2022, respectively. These mild and strong drought conditions corresponded to 10% and 30% less precipitation during the crop growth period recorded in 2021 and 2022 compared to the average for many previous years.

Chickpea seeds of the selected genotypes were included in the field test and sown at the regular scheduled time, in 10–13 May. Plots were 1 m^2^ with a density of 10 plants per m^2^. Each genotype had three replicated plots in a randomized block design. Seeds sown manually were watered once immediately after sowing with no further watering. Seed-related traits were measured individually in each plant after harvesting, as used in other chickpea studies (Purdy et al., 2023; Tiwari et al., 2023). For the purpose of this study, only two traits were considered as follows: seed weight per plant (SWP) and hundred seed weight (HSW), which was measured and calculated directly for the seeds of each plant.

### Statistical treatment

Excel 365 (Microsoft) and SPSS 25.0.0.0 (IBM) packages were used to calculate and analyze means, standard errors, and significance levels using unpaired *t*-test, ANOVA, and *post-hoc* Tukey test. Three biological and two technical replicates were used for RT-qPCR experiments, whereas two repeats were used for seed yield traits and field trials.

## Results

### Molecular phylogenetic analysis of ZF-CCHC proteins in chickpea and other legumes

**Figure 1:**
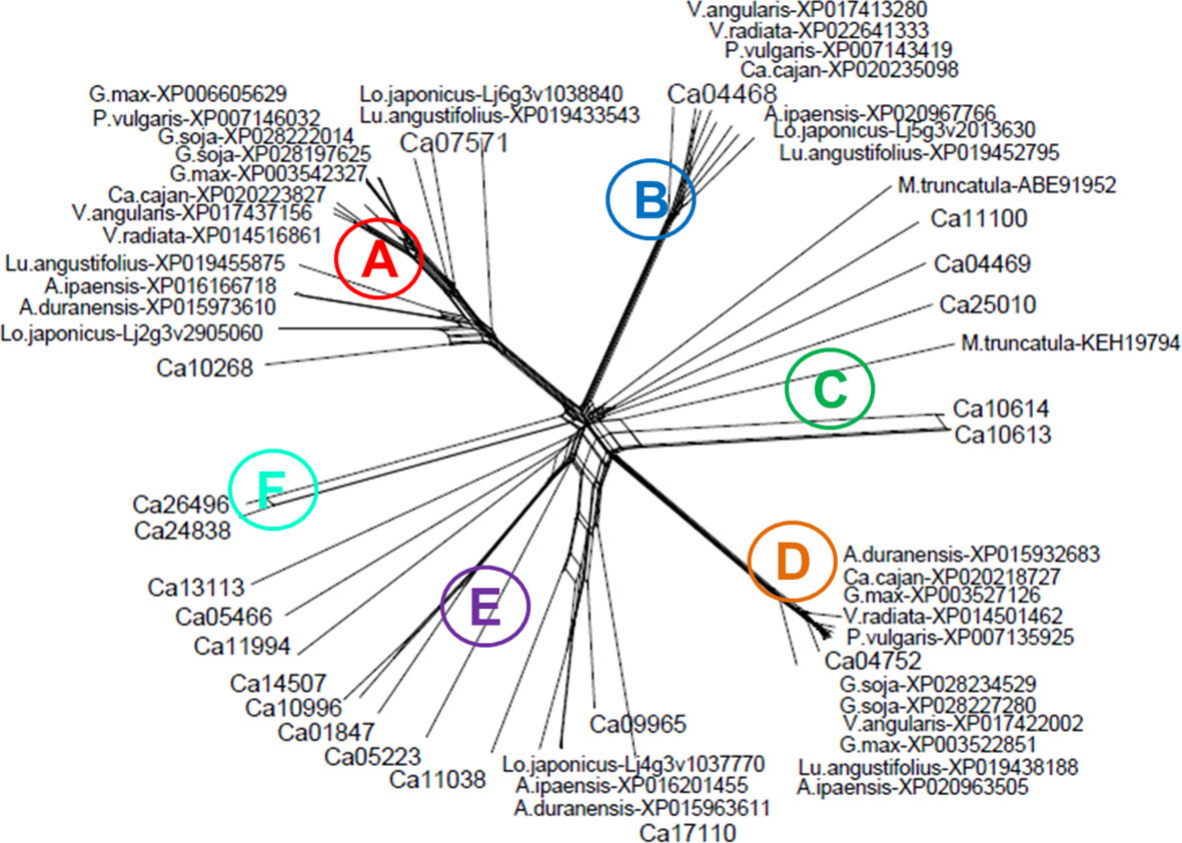
Molecular-phylogenetic tree of zinc finger proteins containing CCHC domains in chickpea and other legume plant species. The chickpea proteins are coded as Ca, *Cicer arietinum* with the following species in alphabetical order: A.duranensis, *Arachis duranensis* (wild ancestor peanut, A genome); A.ipaensis, *Arachis ipaensis* (wild ancestor peanut, B genome); Ca.cajan, *Cajanus cajan* (pigeon pea); G.max, *Glycine max* (soybean); G.soja, *Glycine soja* (wild soybean); Lo.japonicus, *Lotus japonicus* (wild model legume); Lu.angustifolius, *Lupinus angustifolius* (narrowleaf, blue lupine); M.truncatula, *Medicago truncatula* (barrelclover); P.vulgaris, *Phaseolus vulgaris* (common bean); V.angularis, *Vigna angularis* (adzuki bean); V.radiata, *Vigna radiata* (mung bean). The proteins were retrieved from the NCBI database (https://www.ncbi.nlm.nih.gov), and chickpea proteins were converted into those annotated in LIS, the Legume Information System database (https://www.legumeinfo.org). The full sequence of the chickpea and other legume proteins are presented in **Supplementary Material S2**.

Based on bioinformatic analysis for chickpea, 21 zinc finger proteins with a CCHC domain were identified (**Supplementary Material S2**). The molecular-phylogenetic tree was constructed for chickpea and several legume species, generating six clades, A-E (**Figure 1**). Clade A represents a diverse group among legumes but almost all proteins contained six CCHC domains and are annotated as ZF-CCHC protein 7. In chickpea, two genes were identified as belonging to Clade A. The first protein, Ca10268, contains seven CCHC domains while the more typical second protein, Ca07571, had six CCHC domains.

Stronger similarity of ZF-CCHC proteins among legumes was found in Clade B, with all of them containing five CCHC domains. However, the annotations of these proteins (e.g. NCBI, https://www.ncbi.nlm.nih.gov) were quite variable. In chickpea, only a single gene, *Ca4468*, was found in Clade B encoding ZF-CCHC domain protein 9. In contrast, Clade C contained five loosely related chickpea proteins, together with two proteins from *M. truncatula*. Two genes in Clade C, *Ca11100* and *Ca25010*, encoded zinc knuckle proteins with a single CCHC domain, but other genes with three CCHC domains had different annotations and variable functions. Clade D appears isolated, where all proteins in legumes clustered very tightly, including a single chickpea gene *Ca04752* encoding a ZF-protein with nine CCHC domains. All genes in Clade D were annotated as *GIS2*, Glucose inhibition of gluconeogenic growth suppressor 2, and are involved in interaction with DNA.

Clade E contained the largest number of proteins, including several from chickpea, and these were annotated as CSDP, cold shock domain proteins, and GRP, Glycine-rich RNA-binding proteins RZ1. The proteins in this Clade contained a diverse number of CCHC domains with as many as 11 domains in Ca17110 to the more typical five domains in Ca09965, and the rest contained only one or two CCHC domains. The last Clade F contained only two chickpea proteins, Ca24838 and Ca26496, and was identified as a typical Zinc knuckle family protein with two CCHC domains but unrelated to CSP or GRP.

For this study, two chickpea genes, *Ca07571* and *Ca04468*, from Clades A and B, respectively, different in a combination of zinc finger domains, were selected for further analysis and functional characterization representing genes potentially responsive to drought and dehydration (**Figure 1**; **Supplementary Material S2**). The first gene, *Ca07571* (accession: XM_012716319), encoded “Zinc finger CCHC domain-containing protein 7” with six CCHC motifs, 528 aa (accession: XP_004502023), UniProt: A0A1S2YAP2. The gene *Ca07571* was located in the annotated reference genome of chickpea cv. Frontier, Ca5: 40,504,781–40,507,811, and has similarity with two genes in *A. thaliana*, At3g43590 and At5g36240, annotated as “Zinc knuckle (CCHC-type) family protein”.

The second selected gene, *Ca04468* (accession: XM_004496505), encoded “Zinc finger CCHC domain-containing protein 9” with five CCHC motifs, 262 aa (accession: XP_004496562), UniProt: A0A1S2XZY1. The gene was located in the reference genome of chickpea cv. Frontier, Ca4: 12,446,515–12,449,051, and has a similarity with the *A. thaliana* gene, At5g52380, annotated as “Vascular-related NAC-domain protein 6”.

### Sequencing and SNP identification in the promoter regions of two genes, *Ca04468* and *Ca07571*, encoding ZF-CCHC proteins in chickpea

**Figure 2:**
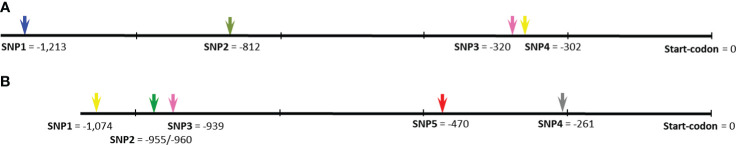
SNP identified in the promoter regions of two *ZF-CCHC* genes, *Ca04468* **(A)** and *Ca07571* **(B)**, in chromosomes Ca4 and Ca5, respectively. The position of each SNP is indicated by the number of nucleotides before the Start-codon. Full description of the identified SNP, corresponding sequences from two reference genomes, cv. Frontier and accession ICC-4958, and their comparisons are present in **Supplementary Material S3**.

Sanger sequencing results for open reading frames (ORF) of both genes, *Ca04468* and *Ca07571*, in six chickpea germplasm accessions as follows: Kamila, Krasnokytsky-123, and Looch (Kabuli type) and ICC-1083, ICC-10945, and ICC-12654 (Desi type) revealed strong conservation, and no SNPs were identified (data not shown). In contrast, promoter regions of both genes contained four SNPs each (**Figure 2**; **Supplementary Material S3**). The distribution of the identified SNP was different in each gene among chickpea accessions in comparison to two reference chickpea genomes cv. Frontier (Kabuli type) and accession ICC-4958 (Desi type).

For gene *Ca04468*, in chromosome Ca4, all four SNP were present in the reference genomes of cv. Frontier and accession ICC-4958 (**Figure 2A**). SNP1 [C/T] was widely distributed among the accessions, while SNP2 [T/C], SNP3 and SNP4 [both A/T] also represented haplotypes for Frontier and ICC-4958, respectively.

For gene *Ca07571*, in chromosome Ca5 (**Figure 2B**), the first three SNPs, at the greatest distance from the Start codon, represented rare alleles and occurred in a single accession only, i.e., SNP1 [A/T] and SNP3 [A/T] in cv. Looch, while a “doubled” SNP2 (two very closely located SNP with just five bp between them), both [T/C], was found only in cv. Kamila. These SNPs were not found in either reference chickpea genome. In contrast, SNP4 [G/A], the most proximally located to the ORF (–261 bp) was equally distributed among six accessions: three Kabuli-type cultivars (Kamila, Krasnokutsky-123, and Looch) had SNP4 [G] similar to reference cv. Frontier, while SNP4 [A], as in reference genome of ICC-4958, was also identified in three Desi-type accessions (ICC-1083, ICC-10945, and ICC-12654). Additionally, SNP5 [T/C] was common between the reference genomes of cv. Frontier and accession ICC-4958, but all six studied chickpea germplasms were monomorphic for SNP5 [C] (**Figure 2B**).

**Figure 3:**
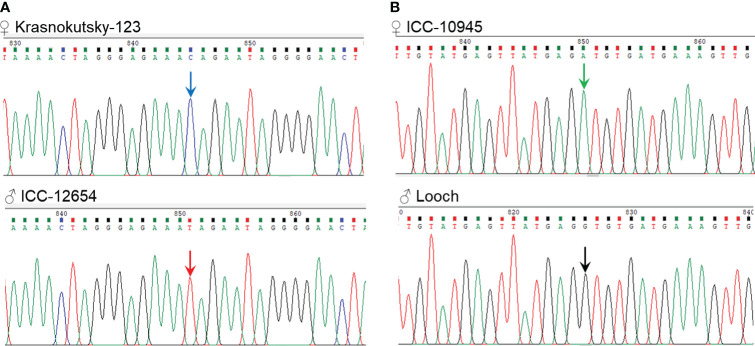
Fragments of Sanger sequencing with two SNPs selected for further study. **(A)** SNP1 [C/T] in gene *Ca04468* in the parents of hybrid population 1, ♀ Krasnokutsky-123 and ♂ ICC-12654, and **(B)** SNP4 [A/G] in gene *Ca07571* in the parents of hybrid population 2, ♀ ICC-10945 and ♂ Looch. The SNP is designated by arrows in the corresponding color.

Finally, one SNP was selected for each gene and confirmed in the parents of two hybrid populations as follows: *Ca04468*-SNP1 [C/T] was suitable for parents, ♀ Krasnokutsky-123 and ♂ ICC-12654, labeled as hybrid population 1, while *Ca07571*-SNP4 [A/G] was selected for the other parents, ♀ ICC-10945 and ♂ Looch, hybrid population 2 (**Figure 3**).

### SNP genotyping of chickpea plants using the ASQ method for two targeted genes, *Ca04468* and *Ca07571*

Genotyping of chickpea plants was carried out based on SNP in each targeted gene, *Ca04468*-SNP1 and *Ca07571*-SNP4, as described above. Parents and breeding lines developed from the progenies were examined. For *Ca04468*-SNP1, hybrid population 1 [♀ Krasnokutsky-123 and ♂ ICC-12654] was used, while hybrid population 2 [♀ ICC-10945 × ♂ Looch] was selected for *Ca07571*-SNP4 genotyping. The parents of both hybrid populations were used as reference genotypes for comparison with their breeding lines (**Figure 3**).

**Figure 4:**
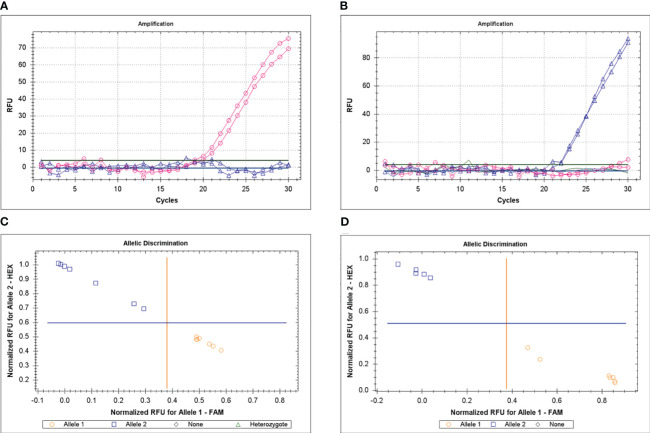
Example of genotyping for *Ca04468* gene in parents, Krasnokutsky-123 and ICC-12654, with FAM **(A)** and HEX **(B)**, respectively, using the ASQ method. Examples of allele discrimination for genes *Ca04468* **(C)** and *Ca07571* **(D)**, respectively, in two hybrid populations, 1 and 2, respectively.

Genotyping was based on the ASQ method and examples of clear amplification of FAM or HEX fluorescence in parents Krasnokutsky-123 or ICC-12654 for *Ca04468* gene are shown (**Figures 4A, B**). Examples of allele discrimination are shown in **Figures 4C, D** for genes *Ca04468* and *Ca07571*, respectively. Results of SNP genotyping for *Ca04468* and *Ca07571* genes are present in **Supplementary Material S4**.

### Validation of SNP genotyping for the *Ca07571* gene using a CAPS marker

The results from SNP genotyping via the ASQ method were confirmed using CAPS, but only for SNP4 in the *Ca07571* gene, because there was no suitable restriction enzyme site targeting the SNP1 fragment in the *Ca04468* gene.

For CAPS marker *Ca07571*-SNP4, a *Mnl*I restriction site was suitable, with the recognition sequence “CCTC” and “GGAG” (in the complemented strain). Due to the presence of several recognition sites in the region surrounding the SNP, a shorter amplification fragment was designed producing a 77-bp digested fragment easily visible for allele [G] in Kabuli-type chickpea (Kamila, Krasnokutsky-123, and Looch). In contrast, there was no such fragment in genotypes with allele [A] in Desi-type chickpea (ICC-1083, ICC-10945, and ICC-12654).

**Figure 5:**
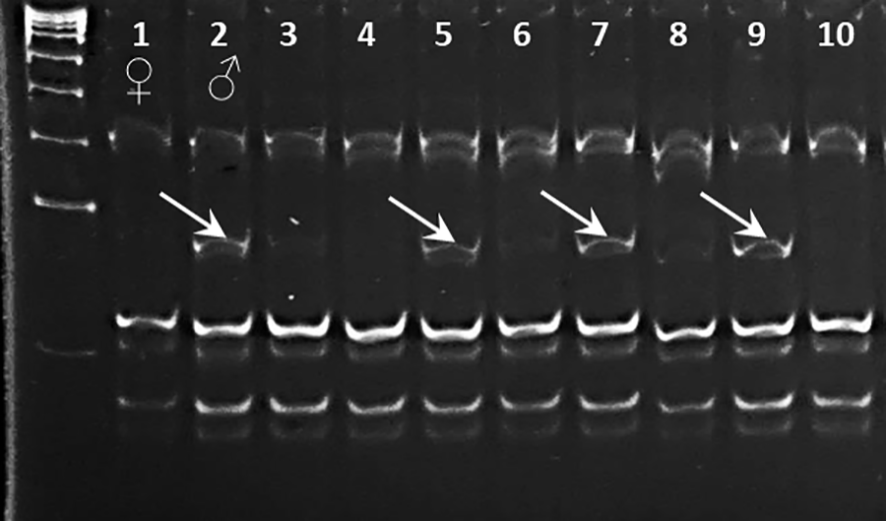
Validation of SNP genotyping for *Ca07571* gene using CAPS marker Ca07571-SNP4-*Mnl*I separated in 12% polyacrylamide gel, in parents (lanes 1 and 2) and selected breeding lines (lanes 3–10) from hybrid population 1 [♀ ICC-10945 × ♂ Looch]. Fragments of 77 bp after digestion with *Mnl*I are indicated by arrows.

Results of CAPS marker analysis of ICC-10945 and Looch, parents of hybrid population 2, and eight of their breeding lines are shown in **Figure 5** after separation either in agarose or in polyacrylamide gels. The paternal genotype Looch and three breeding lines confirmed the [G] allele in *Ca07571*-SNP4.

### Analysis of 6K Diversity Array Technology (DArT) markers for haplotype study in *Ca04468* and *Ca07571* genes

**Figure 6:**
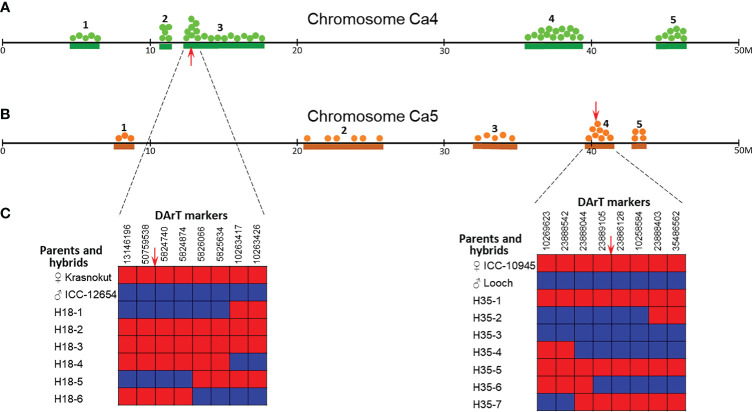
Distributions of DArT haplotype blocks in chromosomes Ca4 **(A)** and Ca5 **(B)** in parents and selected breeding lines from hybrid population 1 (Krasnokutsky-123 and ICC-12654) and hybrid population 2 (ICC-10945 and Looch), respectively. Five identified haplotype blocks with numbers above are designated by green and brown lines according to the position on the physical map of chickpea cv. Frontier, 50M bp in each chromosome. Green and brown dots correspond to SNPs found in parents and their breeding lines. Red arrows indicate positions of the target genes *Ca04468* in chromosome Ca4 **(A)** and *Ca07571* in chromosome Ca5 **(B)**. Representative “zoom” of these genetic regions with both target genes **(C)** shows the distribution of eight surrounding DArT markers in parents and hybrid breeding lines. Maternal and paternal alleles are designated by red and blue boxes, respectively. Sequences and genetic positions of the used DArT markers are present in **Supplementary Material S5**.

DArT-seq analysis was applied to study genetic polymorphism and variability among a set of chickpea accessions and several hybrid populations, including hybrid populations 1 and 2, described above. After an initial filtration of the 6,000 DArT markers, 3,600 of them were polymorphic, and following that, 1,600 DArT markers had known mapping locations in the chickpea genome. For this study, DArT markers identified in the physical map of chromosomes Ca4 and Ca5 cv. Frontier were used for further analysis (**Figure 6**). Sequences and genetic positions of the used DArT markers and raw-data for DArT microarray analysis are present in **Supplementary Materials S5**, **S6**, respectively.

Among 298 DArT markers in chromosome Ca4 with approximately 59M bp in length, five major genetic blocks were found indicating differences in the haplotypes between parents of hybrid population 1, Krasnokutsky-123, and ICC-12654. These DArT haplotype blocks varied in size from 0.22M bp (Block 2) to 5.9M bp (Block 3), and blocks 1–3 and 4–5 were located in opposite arms of chromosome Ca4 (**Figure 6A**).

For chromosome Ca5, which is approximately 69M bp in length, only 176 DArT markers were suitable for the analysis. Haplotypes of ICC-10945 and Looch, parents of hybrid population 2, were also characterized by five major genetic blocks. Blocks 5 and 2 were the shortest and longest DArT haplotype blocks and accounted for about 1M and 5.3M bp, respectively. The five haplotype blocks were distributed relatively similarly along chromosome Ca5 and without a tendency for concentration on the chromosome arms (**Figure 6B**). The density of DArT markers in chromosome Ca5 was fewer by 1.7-fold, compared to chromosome Ca4, and resulted in less identified SNPs in each haplotype block in chromosome Ca5 compared to chromosome Ca4, respectively.

A more detailed zoom of the genetic regions is shown in **Figure 6C**. For gene *Ca04468* in chromosome Ca4, the region between DArT markers 13146196 and 10263426 covered approximately a 1.8M-bp fragment of the chromosome. DArT markers 50759538 and 5824740 were identified as flanking the target gene *Ca04468*. Based on the flanking genetic region, four out of six hybrid breeding lines had a DArT haplotype identical to the maternal parent cv. Krasnokutsky-123. The haplotypes of two breeding lines H18-1 and H18-5 were identical to the paternal parent ICC-12654. All recombination events in the entire genetic region took place in four breeding lines, but all in the proximal part, i.e., between DArT markers 5825634 and 10263417 in lines H18-1 and H18-4, and between DArT markers 5824874 and 5826066 in lines H18-5 and H18-6 (**Figure 6C**).

Similarly, for gene *Ca07571* in chromosome Ca5, the region between DArT markers 10269623 and 35486562 covered a chromosome fragment of approximately 1.9M bp. The flanking DArT markers 23889105 and 23886128 surrounded the target gene *Ca07571*. Three hybrid breeding lines (H35-1, H35-5, and H35-7) had a DArT haplotype identical to the maternal parent ICC-10945. The haplotype of the paternal parent cv. Looch was found in the remaining four hybrid breeding lines. Four breeding lines, H35-2, H35-4, H35-6, and H35-7, have undergone various recombination events in the entire genetic region, both in proximal and distal parts from the target gene *Ca07571* (**Figure 6C**).

For further studies on gene expression, the following genotypes were selected: (1) the first three breeding lines (H18-1, H18-2, and H18-3) from hybrid population 1 together with their parents, Krasnokutsky-123 and ICC-12654; and (2) the first four breeding lines (H35-1, H35-2, H35-3, and H35-4) from hybrid population 2 and their parents, ICC-10945, and Looch.

### RT-qPCR expression analysis of *Ca04468* and *Ca07571* genes in response to drought and dehydration in chickpea parents and breeding lines

**Figure 7:**
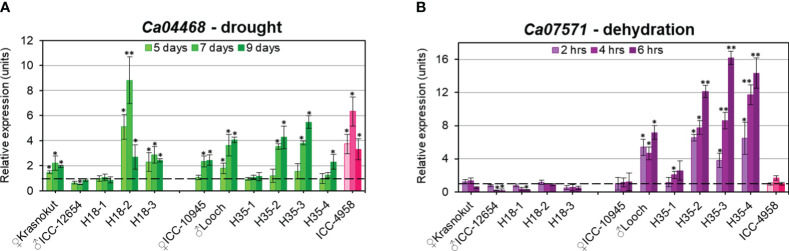
RT-qPCR expression analysis of *Ca04468* under drought stress **(A)** and *Ca07571* after dehydration treatment **(B)** in chickpea plants of parents and selected breeding lines from two hybrid populations. On the left-hand side of each panel, parents Krasnokutsky-123 and ICC-12654 and three breeding lines H18 represent hybrid population 1, while parents ICC-10945 and Looch and four breeding lines H35, on the right-hand side of the panels, belong to hybrid population 2. Plants of ICC-4958 were included as a reference genotype with a fully sequenced genome and are indicated in pink. For drought, leaf samples were collected from soil-grown plants when water was withdrawn, whereas detached leaves were exposed to dehydration on paper-towel at room temperature. Four consecutive time-points were used for sampling, and “point 0” was arranged as controls in both treatments. For all genotypes and experiments, the expressions of controls were set as unit level 1, indicated by dashed lines. Expression data were normalized using two reference genes, *CaELF1α* (elongation factor 1-alfa) and *CaHSP90* (heat shock protein 90), and are present as the average ± SE of three biological replicates (individual plants) and two technical repeats for each genotype and treatment. Significant differences (*p < 0.05 and **p< 0.01) from level 1 were calculated using two-way ANOVA with *post-hoc* Tukey test.

Under drought stress, chickpea plants from the parental accessions and breeding lines from hybrid populations 1 and 2 showed both some similarities and differences. The expression of the *Ca04468* gene was diverse among genotypes in each hybrid population (**Figure 7A**). For example, in parent Krasnokutsky-123, the expression level of *Ca04468* was significantly higher at all time-points of drought treatment compared to Controls. A similar pattern was observed in breeding line H18-3, and it was very high in line H18-2 especially after 7 days of drought stress. In contrast, no changes in *Ca04468* expression under drought was found in parent ICC-12654 and breeding line H18-1. A different situation was found in hybrid population 2, where both parents, ICC-10945 and Looch, as well as two breeding lines, H35-2 and H35-3, showed increased mRNA level and corresponding gene expression. Unexpectedly, no changes in *Ca04468* expression were found in line H35-1, while only delayed increase in expression was recorded in line H35-4 after 9 days of drought treatment. The reference genotype ICC-4958 with a high level of mRNA production was closer to genotypes of Looch and ICC-10945 rather than to ICC-12654 (**Figure 7A**).

The situation was dramatically different when the genes were studied in plants after dehydration treatment (**Figure 7B**). For the *Ca07571* gene, the parents and breeding lines of hybrid population 1 showed constantly low expression and downregulation in some cases. However, the *Ca07571* mRNA level was quite different in the parents and breeding lines from hybrid population 2. The parent ICC-10945 had no change in *Ca07571* expression, while parent Looch and three breeding lines, in contrast, had a high increase in gene expression. The exception is in breeding line H35-1, which showed an increased expression level after 4 h of dehydration, but it was still much smaller than the other three breeding lines in hybrid population 2. The reference genotype ICC-4958 showed no changes in *Ca07571* expression under dehydration, similar to the majority of the studied genotypes, but differing from Looch (**Figure 7B**).

### Seed-related traits in parents and selected breeding lines based on *Ca04468* and *Ca07571* genes

**Figure 8:**
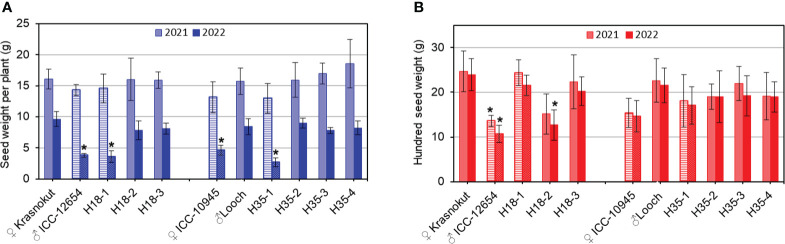
Seed-related traits: seed weight per plant **(A)** and hundred seed weight **(B)** in parents and selected breeding lines from hybrid populations 1 and 2. Data were calculated as the average of the plants grown in the field experiments, 1 m^2^ plot (*n* = 10), with three replicates, in the Akmola region, Northern Kazakhstan, over 2 years, 2021 (lighter color) and 2022 (darker color) with mild and strong drought, respectively. Parents and breeding lines that carried unfavorable alleles of *Ca04468* and *Ca07571* genes are shown in bars with stripes and dots. Error bars represent standard errors. Significant differences (*p < 0.05) are shown for genotypes compared to another parent and breeding lines in each hybrid population, and were calculated using two-way ANOVA with *post-hoc* Tukey test.

The parents and selected breeding lines from hybrid populations 1 and 2 were evaluated for seed-related traits (seed weight per plant, SWP; and 100-seed weight, HSW) in field experiments in Northern Kazakhstan for 2 years under mild and strong drought conditions (**Figure 8**).

SWP did not vary significantly among genotypes during mild drought stress in 2021 regardless of their alleles of both *Ca04468* and *Ca07571* (**Figure 8A**). In contrast, clear discrimination for SWP was found between genotypes in conditions of strong drought in the field study in 2022. Parent ICC-12654 and breeding line H18-1 showed a significantly lower SWP, and they also shared the same allele *Ca04468-*SNP1, which can be described as “unfavorable” for this trait. Other breeding lines H18-2 and H18-3 with *Ca04468-*SNP1 allele identical to the maternal parent Krasnokutsky-123 showed significantly higher SWP. Similarly, in hybrid population 2, a significantly lower SWP was recorded in maternal parent ICC-10945 and one breeding line H35-1. They have the same allele *Ca07571-*SNP4, which is also unfavorable for this trait. Another allele is present in the paternal parent Looch and the remaining three breeding lines H35 with a significantly higher SWP (**Figure 8A**).

Different results were found for HSW, where alleles of *Ca04468* and *Ca07571* were not associated with this trait (**Figure 8B**). In hybrid population 1, the seeds of paternal parent ICC-12654 and breeding line H18-2 showed a significantly smaller HSW, while other genotypes had a significantly higher HSW. In hybrid population 2, no significant differences were recorded for HSW among all genotypes, while the maternal parent ICC-10945 recorded a slightly lower HSW, probably the result of smaller seeds, but not significantly lower compared to other genotypes. The results indicate that alleles of *Ca04468* and *Ca07571* did not affect the HSW trait in chickpea genotypes under conditions of mild and strong drought (**Figure 8B**).

## Discussion

Genes encoding zinc finger proteins with CCHC domains are important in various processes of plant life, but little information is available about their function in chickpea. The two genes selected for this study, *Ca04468* and *Ca07571*, belong to different Clades B and A in a molecular–phylogenetic tree (**Figure 1**). They encode zinc finger CCHC domain-containing proteins 7 and 9 with unknown functions in chickpea plants. Forward and reverse genetic approaches are often used to address this issue. Instead of going from phenotype to sequence as in forward genetics, reverse genetics works in the opposite direction—from known sequence toward assigned gene function (Alonso and Ecker, 2006). However, reverse genetics was used in a different way in our study. We focused on genes described in *Arabidopsis* and other plants species that showed a high degree of similarity to the genes in chickpea, *Ca04468* and *Ca07571*, and these were characterized for the first time in the current research. These two selected candidate genes with a specific combination of Zn finger domains were shown to be expressed in the chickpea genome (**Supplementary Material S2**). Therefore, our research leverages selected genes studied earlier in other plants species for the discovery of novel genes in chickpea; and this method is very different from forward genetics with QTL and linkage analysis (Aklilu, 2021).

However, a confusing situation exists in cluster 6 with wheat and rice ZF-CCHC proteins, where the same or similar proteins have different annotations (Sun et al., 2022). For example, protein LOC_Os04g46920, of 277 aa in length, was described as a “Zinc knuckle domain-containing protein” (Rice Genome Annotation Project: http://rice.uga.edu), while the same protein sequence, NP_001406677 = LOC129456122 = Os04g0555800, was annotated as “Arginine methyltransferase-interacting protein” (NCBI: https://www.ncbi.nlm.nih.gov). Furthermore, the closest homolog in *A. thaliana*, At5g52380, was annotated as “Vascular-related NAC-domain 6,” 268 aa in length (Yamaguchi et al., 2008). Such dissimilar protein descriptions reflect the complexity and wide diversity of ZF-CCHC proteins, including those containing NAC-domains or interactivity with arginine methyltransferase, perhaps relating to different motifs and structural elements within the proteins.

The other chickpea *ZF-CCHC* genes do not have clear similarity with wheat and rice homologs. For example, genes from Clade E in chickpea (cold shock proteins and glycine-rich proteins with CCHC domains) were similar to several clusters in wheat and rice, including clusters 7 (CSP) and 1–3 (GRP), respectively (**Figure 1**). Proteins in Clade C had altogether weak similarity with ZF-CCHC proteins in clusters with wheat and rice (Sun et al., 2022).

Based on the ORF sequences of the two selected genes, *Ca04468* and *Ca07571*, their encoded proteins were highly conserved. Nevertheless, our results demonstrate significant genetic polymorphism in the promoter regions of several chickpea accessions (**Figures 2**, **3**). This could indicate that the regulation of *Ca04468* and *Ca07571* genes occurs via additional transcription factors differentially binding the promoters of these genes to enhance or inhibit their expression. However, there is no evidence for this yet, but this may be a promising area for future experiments, where potential transcriptional factors regulating the studied *ZF-CCHC* genes may be identified using, for example, RNA-seq technology. Any SNP in coding or non-coding regions of genes can be used for molecular marker development and further plant genotyping, as demonstrated by previous successful applications for molecular breeding in chickpea (Hiremath et al., 2012; Kujur et al., 2015; Shimray et al., 2017), including marker KATU-C22 developed by the authors (Khassanova et al., 2019). In the current study, suitable SNPs, *Ca04468*-SNP1 and *Ca07571*-SNP4, were identified in promoter regions of the genes (**Figures 2**, **3**).

In the current study, the ASQ method was adapted and successfully used for the first time for the genotyping of chickpea plants. In the established hybrid populations, two parents (vs. Krasnokutsky-123 and Looch), known to be well-adapted to the dry environments of Kazakhstan, were crossed with germplasm accessions with superior seed quality and protein content. Based on plant genotyping performed using the ASQ method and verified via CAPS markers, parents and hybrid breeding lines were assessed for alleles of *Ca04468-*SNP1 and *Ca07571-*SNP4 in two *CaZF-CCHC* genes in chickpea. The ASQ method based on fluorescence measurement showed strong amplification of either FAM or HEX signals (**Figure 4**). The allele discrimination and genotyping results are clear using this ASQ method with medium throughput working effectively in 96- or 384-well microplates and qPCR instruments. Successful results for the ASQ method were recorded earlier in the genotyping of barley (Kalendar et al., 2022), sugar beet, and tomato (Amangeldiyeva et al., 2023).

CAPS markers are useful for the verification of genotyping results. However, the CAPS method has also significant limitations. For example, only one marker, *Ca07571*-SNP4-*Mnl*I, was developed with a single restriction enzyme. In the current study, this CAPS marker was generated and used, but the application of this CAPS marker was much slower and more expensive. For the other marker, *Ca04468*-SNP1, there was no restriction enzyme available with a recognition site targeting the SNP. In this situation, it may have been possible to develop a dCAPS marker (derived CAPS), where a restriction site can be introduced at the SNP position using specific primers. However, this additional step further complicates the process. In a comparison between both methods, ASQ genotyping definitely has an advantage. Nevertheless, in chickpea research, both CAPS and dCAPS markers have also been used effectively (Gujaria et al., 2011; Jaganathan et al., 2015). They remain slower and more expensive compared to other modern methods of plant genotyping.

DArT-seq microarray analysis is a very different and powerful approach. Our results demonstrated that the genetic regions with the identified SNPs were included in the DArT-based haplotype groups. Therefore, microarray and DArT markers confirmed the genotyping results for the two genes studied using an alternative technology. A similar DArT method was used for the mapping and identification of genetic regions with candidate genes in chickpea hybrid populations (Thudi et al., 2011; Atieno et al., 2021).

RT-qPCR analysis revealed that the gene *Ca04468* was strongly expressed in both hybrid populations under slowly developed drought, and it was associated with *Ca04468-*SNP1 genotypes in parents and breeding lines of hybrid population 1. In contrast, under rapid dehydration, no change, or downregulation of *Ca04468*, gene expression was evident in genotypes of hybrid population 1, while a strong association was found in parents and breeding lines of hybrid population 2 associated with *Ca07571*-SNP4 genotypes exposed to dehydration. Therefore, both genes, *Ca04468* and *Ca07571*, were specific in their expression to drought and dehydration, and genotype-dependent in hybrid populations 1 and 2, respectively.

The expression analysis of the two studied genes was associated with genotyping results using ASQ markers, *Ca04468*-SNP1 and *Ca07571*-SNP4. These markers were developed based on SNP in promoter regions of the genes, and at the current stage, we can hypothesize that chickpea haplotypes with different SNPs can confer the regulation of gene expression in plants under drought and dehydration. Additionally, in further experiments, the two ASQ molecular markers, *Ca04468-*SNP1 and *Ca07571*-SNP4, developed for the studied genes, must be tested in broader application in chickpea breeding programs, with analysis of their advantages and potential limitations.

Finally, from seed-related traits, SWP was strongly associated with *Ca04468* expression in plants of hybrid population 1, while a similar significant association with *Ca07571* was found in hybrid population 2. This indicates that both genes play an important role in plants grown under mild and especially severe drought conditions. However, their mechanisms of action differed in soil-based drought and under dehydration in detached leaves. These results are similar to many others published earlier during comparisons of the slow drying of soil or substrate, where plants can adapt to drought gradually, or dehydration shock, with the sudden detaching of leaves or removal of plants from hydroponics. For example, drought and dehydration have different effects for aquaporin genes in resurrection plants (*Craterostigma plantagineum* Hochst.) (Mariaux et al., 1998), two genes encoding enzymes of the abscisic acid biosynthesis pathway in tomato (*Lycopersicon esculentum* Mill.) (Thompson et al., 2000), ascorbate peroxidase genes in cowpea [*Vigna unguiculata* (L.) Walp.] (D’Arcy-Lameta et al., 2006), dehydrin genes in Bermuda grass [*Cynodon dactylon* (L.) Pers.] (Hu et al., 2010), and several antioxidant-related genes and transcription factors in barley (*Hordeum vulgae* L.) (Gürel et al., 2016). It can be concluded that experiments with drought and dehydration must be considered very differently.

In our study, seeds were analyzed in individual plants grown in real field experiment under different levels of drought, and this method is used often in chickpea (Chetariya et al., 2022; Patil et al., 2023; Sundaram et al., 2023). Seed-related traits are complex (Sundaram et al., 2018), where many genes are involved, including chickpea (Shimray et al., 2017; Kivrak et al., 2020). For the SWP trait, *Ca09705*, an ABC transporter gene for glutathione conjugates was reported and mapped to chromosome Ca2 in chickpea collections and hybrid populations (Basu et al., 2019). The authors reported an enormous 20% increase in seed yield in hybrid plants with favorable haplotypes of the *CaABC* transporter gene under growing conditions without drought or dehydration. In the same chromosome Ca2, another gene *Ca03044*, encoding a pentatricopeptide repeat (PPR)-containing protein, was closely associated with SWP in chickpea using genome-wide genotyping of informative SNPs under non-stressed conditions (Basu et al., 2018).

Seed number per pod was not analyzed in this study, but it ultimately relates to seed yield in chickpea, and was regulated by the *Ca17942* gene, cellulose synthase, *CesA3*, mapped to chromosome Ca5 (Kujur et al., 2015). It was found during genome-wide SNP scanning for strong association in a chickpea grown without stresses. In proteomics analysis, drought-tolerant and sensitive chickpea genotypes showed changes in 29 and 30 proteins, respectively, under drought stress (Vessal et al., 2020). Such massive changes need careful analysis of the identified proteins and encoding genes. Among more than 430K studied SNPs, the NAC transcription factor (*Ca05696*, chromosome Ca6) and possible downregulated gene-encoded histone H3 protein (*Ca15001*, chromosome Ca2) harbor the major QTL for seed yield under drought in chickpea (Sharma et al., 2019). Therefore, many genes have been identified as involved in seed yield in chickpea, both in normal conditions and under drought; in our study, *CaZF-CCHC* genes for the first time showed some promising results for seed weight per plant grown in a dry environment. Additionally, phenotypic variability of seed-related traits and tolerance to drought and dehydration must be studied and verified in different chickpea accessions and breeding lines grown in diverse environments in future experiments.

No associations between *ZF-CCHC* genes and seed yield in plant species have been published to our knowledge. However, in an interspecific cross between bread wheat and *Triticum spelta* (3338 × Di7), the glycine-rich protein with RNA-binding and CCHC domains, AG5, was very strongly indicated in young leaves of F_1_ hybrid plants compared to both parents (Ni et al., 2000). These hybrid plants showed 40% more grain yield compared to the high-yielding wheat parent 3338. The authors hypothesized about the *AG5* gene role in wheat heterosis, but they also indicated that many other genes were expressed specifically in this hybrid (Ni et al., 2000). The *AG5* was reported to have a strong similarity to the *RZ1* gene in tobacco and belongs to cluster 3 in the wheat study by Sun et al. (2022). We identified from NCBI, the wheat protein, accession AAK01176, identical to AG5 and annotated it as “RNA-binding protein with zinc knuckle CCHC domain,” with the corresponding gene accession AF315811. BLAST comparison revealed that the closest homolog of AG5 protein in chickpea was Ca05223 (accession: XP004504758), which is located in Clade E (**Figure 1**). This discovery indicates the very diverse functions of *ZF-CCHC* genes in different crop species, and each candidate gene needs careful evaluation in future analysis.

In our study, both genes, *Ca04468* and *Ca07571*, showed no influence on HSW. These results differed from those published earlier for chickpea, where two genes, *Ca04364* and *Ca04607*, were identified as strongly associated with HSW. These genes were mapped to chromosome Ca4 and encoded a cell division protein kinase and a transmembrane protein, respectively (Singh et al., 2016), and a very different chickpea hybrid population was used in that study [ICC 4958 × ICC1882].

The HSW trait is presumably directly related to seed size. Our findings were also different from those for the *MtZF-CCHC* gene in *Medicago truncatula*, where seed size was significantly enlarged in plants with overexpression of this gene (Radkova et al., 2019, Radkova et al., 2021). However, the MtZF-CCHC protein was similar to those in Clade C presented here (**Figure 1**) and very different from Clades A and B including the chickpea proteins encoded by *Ca04468* and *Ca07571*.

In summary, the two genes, *Ca04468* and *Ca07571*, which code for zinc finger knuckle motifs with CCHC domains, were identified as potential important candidate genes associated with plant response to drought and dehydration. The SNP-based genotypes had different roles in traits of seed weight per plant but not 100-seed weight. The developed and verified two SNP molecular markers for both genes, *Ca04468* and *Ca07571*, could be used for marker-assisted selection for improving tolerance to drought and dehydration and may be applied to the production of novel chickpea cultivars in the future.

## Data availability statement

The original contributions presented in the study are included in the article/**Supplementary Material**, further inquiries can be directed to the corresponding authors.

## Author contributions

GK: Investigation, Writing – original draft. IO: Data curation, Writing – review & editing. ET: Software, Writing – review & editing. SJ: Funding acquisition, Writing – review & editing. NZ: Validation, Writing – review & editing. AG: Visualization, Writing – review & editing. NG: Writing – review & editing, Resources. CSc: Writing – review & editing, Writing – original draft. AP: Resources, Writing – review & editing. LP-D: Visualization, Writing – review & editing. PA: Methodology, Writing – review & editing. CSw: Formal analysis, Writing – review & editing. CJ: Funding acquisition, Writing – review & editing. KS: Supervision, Writing – review & editing. YS: Conceptualization, Writing – original draft.

## Supplementary material

Supplementary Material S1 Sequences and information about primers and molecular probes used for: (1) Sequencing of fragments in coding regions and promoters; (2) ASQ genotyping; (3) CAPS markers; (4) RT-qPCR gene specific and reference gene primers.

Supplementary Material S2 Sequences and information about 21 Zinc-finger proteins with CCHC domain in chickpea as similar genes in legumes.

Supplementary Material S3 Promoter regions of two studied genes, *Ca04468* and *Ca07571*, with identified SNP in chickpea.

Supplementary Material S4 Results of SNP genotyping of *Ca04468* and *Ca07571* genes using ASQ method.

Supplementary Material S5 Sequences and genetic positions of the used DArT markers for the analysis of *Ca04468* and *Ca07571* genes.

Supplementary Material S6 The raw-data of DArT microarray analysis.
