# Supplementary material for: Zinc finger knuckle genes are associated with tolerance to drought and dehydration in chickpea (Cicer arietinum L.)
Source: Front Plant Sci. 2024 May 3;15:1354413. doi: 10.3389/fpls.2024.1354413 (PMC11099236; doi:10.3389/fpls.2024.1354413)
Supplement: Supplementary Material S1 — Sequences and information about primers and molecular probes used for: (1) Sequencing of fragments in coding regions and promoters; (2) ASQ genotyping; (3) CAPS markers; (4) RT-qPCR gene specific and reference gene primers. [file DataSheet_1.zip › Khassanova-Supplementary-material-S1-S5-26-04-2024.pdf]

Supplementary Material

Supplementary material S1. Sequences and information about primers and molecular probes used for: (1) Sequencing of fragments in coding regions and (2) promoters; (3) ASQ genotyping; (4) CAPS markers; (5) RT-qPCR gene specific and reference gene primers.

Name	Sequence 5'-3'	Amplicon size (bp)
**1. Primers for sequencing in coding regions**		
Ca07571-seq-F1	AACTCTTTGCGGCATAGGAAGG	1,131
Ca07571-seq-R1	GGTGGCCACCTTTCTTACAGAT	
Ca07571-seq-F2	ATCTGTAAGAAAGGTGGCCACC	1,062
Ca07571-seq-R2	TCTCGCTTTGCAACCTTGAGCA	
Ca07571-seq-F3	TGCTCAAGGTTGCAAAGCGAGA	928
Ca07571-seq-R3	GAACACGAAGGCGAAAGCTACT	
Ca04468-seq-F1	TGAACCCACTCCTCCTAAAGAC	1,165
Ca04468-seq-R1	ACAGTACATCTCCACTACCGGA	
Ca04468-seq-F2	TCCGGTAGTGGAGATGTACTGT	1,103
Ca04468-seq-R2	AGCACCCTTCTACCAGATGACT	
**2. Primers for sequencing in promoter regions**		
Ca07571-seq-F4	GCAATAATCTGCACGCCTTCCT	1,206
Ca07571-seq-R4	ACATTTGAGGGGTGTGGGTAGT	
Ca07571-seq-F5	ACTACCCACACCCCTCAAATGT	1,101
Ca07571-seq-R5	CCTTCCTATGCCGCAAAGAGTT	
Ca04468-seq-F3	CTAGGGAGAAACAGAATAGGGGA	982
Ca04468-seq-R3	GTATACAGCGCGGACCTTTTCA	
Ca04468-seq-F4	CAGAGGCACATGGGTTGAGAAT	1,089
Ca04468-seq-R4	GACAAGATGTGGCCGATGATTG	
Ca04468-seq-F5	TCAACACTCTCAAACCCGTCTC	1,206
Ca04468-seq-R5	TCAACCCATGTGCCTCTGTATC	
**3. Primers and molecular probes for ASQ genotyping**		
Ca07571-SNP4-F1	GTCCTTGCGAAGGCATCCCTCACATTTATCAACTTTCATCACACC	120
Ca07571-SNP4-F2	GTCCTTGCGAAGGCCAACCTCACATTTATCAACTTTCATCACATC	
Ca07571-SNP4-R	CTGAGTTCACGTAAAATACCCAGAT	
Ca04468-SNP1-F1	GTCCTTGCGAAGGCATCCGTGGATTAAAACTAGGGAGAAATA	87
Ca04468-SNP1-F2	GTCCTTGCGAAGGCCAACGTGGATTAAAACTAGGGAGAAACA	
Ca04468-SNP1-R	AAGGCTTAATTGCAGTTTTGATCC	
UP1-FAM	FAM-GTCCTTGCGAAGGCATCC	-
UP2-HEX	HEX-GTCCTTGCGAAGGCCAAC	
Uni-Q	GCCTTCGCAAGGAC-Dabcyl	
**4. Primers for CAPS**		
Ca07571-CAPS-F	CTGAGTTCACGTAAAATACCCAGA	162
Ca07571-CAPS-R	AATGCCCCTGCCATCAATGTTA	

5. Primers for RT-qPCR		
Ca07571-Fq	GTCCAAAAGGAGCAGTTGGAGG	149
Ca07571-Rq	GGTAATTTGAGGCTCCAGGTTG	
Ca04468-Fq	TGACACATCTGGCAAGGGATTG	137
Ca04468-Rq	CATATCGTCTCCGCTCACAAAC	
CaELF1-Fq	TCCACCACTTGGTCGTTTTG	64
CaELF1-Rq	TTAATGACACCGACAGCAACAG	
CaHSP90-Fq	GCAGCATGGCTGGTTACATGT	63
CaHSP90-Rq	TGATGGGATTCTCAGGGTTGA	

Supplementary material S2. Sequences and information about 21 Zinc-finger proteins with CCHC domain in chickpea as similar genes in legumes.

Information about 21 chickpea accessions, in clockwise order occurring in the phylogenetic tree, number of CCHC motives, gene names, position in the genome of reference cv. Frontier, and homologs in Arabidopsis.

1. A1-Ca10268 (431aa) = similar to XP004491141 (XM_004491084), 4 CCHC motives.
Ca_10268.1 locus=Ca2:32544020:32547949. Expressed.
At3g43590 = Zinc knuckle (CCHC-type) family protein.

2. A2-Ca07571 (528aa) = XP004502023 (XM_012716319), 6 CCHC motives; Zinc finger CCHC domain-containing protein 7, UniProt: A0A1S2YAP2.
Ca_07571.1 locus=Ca5:40,504,781:40,507,811; Expressed.
At3g43590 + At5g36240 = Zinc knuckle (CCHC-type) family protein.

3. B-Ca04468 = similar to XP004496562 (XM_004496505), 3 CCHC motives; Zinc finger CCHC domain-containing protein 9.
Ca_04468.1 locus=Ca4: 12,446,515:12,449,051. Expressed – shoots and flowers.
At5g52380 = Vascular-related NAC-domain protein 6.

4. C1-Ca11100 – 1 CCHC motif.
Ca_11100.1 locus=Ca6:23548581:23550829. Expressed in buds and flowers.
At3g02820 = Zinc knuckle (CCHC-type) family protein.

5. C2-Ca04469 – 3 CCHC motives.
Ca_04469.1 locus=Ca4:12452671:12471086. Expressed – young leaves and flowers.
At4g25590 = ADF7, actin depolymerizing factor 7.

6. C3-Ca25010 – 1 CCHC motif.
Ca_25010.1 locus=scaffold2166:62542:63258. Expressed – mostly in flowers.
At3g10400 = RNA recognition motif and CCHC-type zinc finger domains containing protein.

7. C4-Ca10614 – 3 CCHC motives.
Ca_10614.1 locus=Ca8:7724822:7728244. Expressed during germination.
At5g45400 = RPA70C, ATRPA70C, Replication factor-A protein 1-related.

8. C5-Ca10613 – 1 CCHC motif.
Ca_10613.1 locus=Ca8:7729646:7729861. Non-expressed.
No hit but similar to previous Ca10614.

9. D-Ca04752 = XP004500771 (XM_004500714) = 9 CCHC motives (GIS).
Ca_04752.1 locus=Ca5:30595088:30597124. Expressed in leaves and buds.
At1g75560 = Zinc knuckle (CCHC-type) family protein.

10. E1-Ca17110 = XP004499769 = 11 CCHC motives.
Ca_17110.1 locus=Ca5:11863958:11874745. Expressed in leaves.
At1g65440 = GTB1, Global transcription factor group B1 and At4g36020 and At3g42860 = CSDP1, Cold shock domain protein 1; and Zinc knuckle (CCHC-type) family protein.

11. E2-Ca09965 = XP004509364 (XM_004509307) = 5 CCHC motives.
Ca_09965.1 locus=Ca7:14834011:14834325. Expressed in leaves.
At4g36020 = CSDP1, Cold shock domain protein 1 and At2g17870 = ATCSP3, CSP3, Cold shock domain protein 3.

12. E3-Ca11038 – 2 CCHC motives.
Ca_11038.1 locus=Ca6:24255868:24256485. Expressed in leaves.
At2g21060 = ATGRP2B, ATCSP4, GRP2B, Glycine-rich protein 2B and At2g17870 = ATCSP3, CSP3, Cold shock domain protein 3.

13. E4-Ca05223 – Similar to wheat AG5 protein. 1 CCHC motif.
Ca_05223.1 locus=Ca6:14527472:14528402. Expressed in leaves.
At3g26420 = ATRZ-1A, RNA-binding (RRM/RBD/RNP motifs) family protein with retrovirus zinc finger-like domain.

14. E5-Ca01847 – 1 CCHC motif.
Ca_01847.1 locus=Ca5:34034638:34036306. Expressed in buds.
At2g24590 = RSZ22a, At-RSZ22a, RNA recognition motif and CCHC-type zinc finger domains containing protein.

15. E6-Ca10996 – 1 CCHC motif.
Ca_10996.1 locus=Ca4:41124549:41127116. Expressed in leaves and buds.
At2g24590 = RSZ22a, At-RSZ22a, RNA recognition motif and CCHC-type zinc finger domains containing protein and At4g31580 = SRZ-22, SRZ22, RSZP22, RSZ22, At-RSZ22, Serine/arginine-rich 22.

16. E7-Ca14507 – 1 CCHC motif.
Ca_14507.1 locus=Ca7:21488620:21491015. Expressed in buds.
At1g23860 = SRZ-21, SRZ21, RSZ21, At-RSZ21, RS-containing zinc finger protein 21; and At4g31580 = SRZ-22, SRZ22, RSZP22, RSZ22, At-RSZ22, Serine/arginine-rich 22.

17. E8-Ca11994 – 2 CCHC motives.
Ca_11994.1 locus=Ca3:32660235:32663521. Expressed in leaves.
At2g37340 = RSZ33, ATRSZ33, RS2Z33, AT-RS2Z33, Arginine/serine-rich zinc knuckle-containing protein 33; and At3g53500 = RSZ32, RS2Z32, At-RS2Z, RNA-binding (RRM/RBD/RNP motifs) family protein with retrovirus zinc finger-like domain.

18. E9-Ca05466 – 1 CCHC motif.
Ca_05466.1 locus=Ca4:16578088:16579240. Expressed in leaves.
At1g60650 = RNA-binding (RRM/RBD/RNP motifs) family protein with retrovirus zinc finger-like domain; and At5g04280 = RNA-binding (RRM/RBD/RNP motifs) family protein with retrovirus zinc finger-like domain.

19. E10-Ca13113 – 1 CCHC motif.
Ca_13113.1 locus=Ca4:38448139:38457150. Expressed in apical meristems.
At1g53720 = ATCYP59, CYP59, cyclophilin 59.

20. F1-Ca24838 – 2 CCHC motives.
Ca_24838.1 locus=Ca6:11838040:11838915. Expressed – mostly in leaves.
At3g42860 and At1g75560 = Zinc knuckle (CCHC-type) family protein.

21. F2-Ca26496 – 2 CCHC motives.
Ca_26496.1 locus=scaffold1626:143832:144554. Expressed in flowers only.
At3g42860 and At1g75560 = Zinc knuckle (CCHC-type) family protein.

Supplementary material S2 (continuation). Protein sequence of legume accessions, annotated information, in alphabetical order. CCHC motives are indicated in yellow. Chickpea accessions are indicated by green.

Abbreviations for plant species are as follows: **A.duranensis**, *Arachis duranensis* (wild ancestor peanut, A genome); **A.ipaensis**, *Arachis ipaensis* (wild ancestor peanut, B genome); **Ca.cajan**, *Cajanus cajan* (pigeon pea); **Ca**, *Cicer arietinum*; **G.max**, *Glycine max* (soybean); **G.soja**, *Glycine soja* (wild soybean); **Lo.japonicus**, *Lotus japonicus* (wild model legume); **Lu.angustifolius**, *Lupinus angustifolius* (narrowleaf, blue lupine); **M.truncatula**, *Medicago truncatula* (barrelclover); **P.vulgaris**, *Phaseolus vulgaris* (common bean); **V.angularis**, *Vigna angularis* (adzuki bean); **V.radiata**, *Vigna radiata* (mung bean).

>A.duranensis-XP015973610, 6 CCHC, protein AIR2
MGRKDKQRAKKTEHEEEEEHGSPMGSTPPLVFEVSSDDEEANEDLSLGIVEKALMRRKLPRNDVVSN
GDDAIILGASSSRQDEVAVARNEGVLNEAREVVDVSDSEELKSERKKKSKSKKKKVKKVESEEHNVV
TAEGQVTIETVETTKPIKEAENDEPAEEPAMVQMGDNAVLRKLLRGPRYFDAPDSGGWGACYNCGEE
GHAAVNCTAAKRKKPCYVCGSLEHNAKHCTMGRDCYICKKGGHRAKDCPEKNLIGSQSLKICLKCGD
SGHEMFSCKNDYSPDDLKEVQCYVCKKFGHLCCVNTADSTPRVISCYQCGQLGHTGLACARLRTEAA
DAATPSSCYRCGEAGHFARECTSSVKLGKRRRESSNTKTPKFQKENDYVGHRSAPHDIGKSWKKKKP
FTEEKGLTTPRKPKHRGGWMTEHPAEFSPSKSKRSSWRSPATPPYKSSKVHSFRNESYTPKSNSSKI
RKFHDPSPTPRRSPHSYQTRFSASRFGHSSSDGYGRSYNWW

>A.duranensis-XP015963611, 6 CCHC, cold shock domain-containing protein 3
MAEERYSGVVQWFSNSKGFGFIKPDQGGDDLFVHHSSIQSDGAFRTLVDGDRVEFSIADNSDKPKAL
DVTGPNGAPLRSAHDSARAGNDNRRSAGAGGGGGGACYQCGDFGHLARDCSRAGNGGGGGGGACY
SCGGFGHMARDCVSGGGGGGANGGGCFKCGEFGHMARDCSGGGGGGGGNGGGCFKCGEFGHMARDCS
GGGGNGGGCYRCGEVGHLARDCNREGGSGAGGNGGKSTCFNCGKPGHFARECVEASG

>A.duranensis-XP015932683, 9 CCHC, zinc finger protein GIS2
MSSDSRSRSRSRSRSRSPMDRKIRSDRFSYRDAPYRRDSRRGFSRDNLCKNCKRPGHYARECPNVAICH
NCGLPGHIASECTTKSLCWNCKEPGHVASNCPNEGICHTCGKVGHRARECTAPPMPPGDLRLCNNCY
KQGHIAAECTNEKACNNCRKTGHLARDCPNDPICNLCNVSGHVARQCPKANMLGDRGTSGGMRGGGG
GYRDVVCRNCQQLGHMSRDCMGPLMICHNCGGRGHLAYECPSGRFVDRYPRRY

>A.ipaensis-XP016166718, 6 CCHC, protein AIR2
MGRKDKQRAKKTEHEEEEEHGSPMGSTPPLVFEVSSDDEEANEDLSLRIVEKALMRRKLPRNDVDSN
GDDAIILGGSSSRQDEVAVARNEGALNEAREVVDVSDSEELKSERKKKSKSKKKKVKKVESEEHNVV
TAEGQVTIETVETTEPIKEAEKDEPAEEPAMVQMGDNAVLRKLLRGPRYFDAPDSGGWGTCYNCGEE
GHAAVNCTAAKRKKPCYVCGSLEHYAKHCTMGRDCYICKKGGHRAKDCPEKNLIGSQSLNICLKCGD
SGHEMFSCKNDYSPDDLKEVQCYVCKKFGHLCCVNTADSTPRVISCYQCGQLGHTGLACARLRTEAA
DAATPSSCYRCGEAGHFARECTSSVKLGKRRRESSNTKTPKFQKENDYVGHRSAPHDIGKSWKKKKP
FTEEKGLTTPRKPKHRGGWMTEHPAEFSPSKPKRSSWRSPATPPYKSSKVHSFRNESYTPKSNSSKM
RKFHDPSPTPRRSPHSYQTRFSASRFGHSNSDGYGRSYNWW

>A.ipaensis-XP020967766, 5 CCHC, uncharacterized protein LOC107616096
MVSQRQRLARKRFKEENPELFPKPEPTPPKDPDKKKKKKKSNTFKSSSIKRRSDGSEELGVSKKPFK
SNYRKHPLRVPGMKPGDTCFICKAADHIAKSCPQKAEWEKNKICLRCRRRGHRAKNCPEVQVGAKDD
KYCYNCGETGHSLANCPHPVQEGGTKFAECFVCKQQGHLSKNCPQNAHGIYPKGGCCKICGGVTHLA
RDCPEKGKKAPFAANGPADGSMRLRNEQRPCGTITKFVSGDDIEDDFMTDDINNRDKDKSSKSKDGQ
AKPKNKGPKVVNFSRSRDDHSNPNPLSSPQHTTTNNKKRRRKHNHAPTPQPPTHRHRYNTRPKANPN
PNPNSGSAVVQQLKETGAENPQLFSKHELAAKLVDLAGKENGQGKYEGAALQLEPEEKLSKNARRRR
KNKNKNNALNNEVAEAEPTKHNHKNKEKKKSKRLLRSNVSESNNYNSESTDEIPLRTGNANSPHHTR
SQGTKALLTGVAYDECRCVNHAKCLVINSGNVRRGKVDTFTRKFASFVGNSVTRLQNARFLQQGEDD
LLGAYCVLPMDTSLISVHKLVMESANAASGMAVNDACKLKFLELKAKRNYRFIVFRIENQEVVVEKV
GSPDETYDDFTASLPANECRYAVFDFDFTTDENCQKSKIFFIAWAPDTSRVREKMVYASSKDRFKRE
LDGIQVELQATDPSEMSFDIIKARAI

>A.ipaensis-XP020963505, 9 CCHC, zinc finger protein GIS2
MAAYAGIRHCLGELAYVDKGHIQYLAILHTRIRSRDNLCKNCKRPGHYARECPNVAICHNCGLPGHI
ASECTTKSLCWNCKEPGHVASNCPNEGICHTCGKVGHRARECTAPPMPPGDLRLCNNCYKQGHIAAE
CTNEKACNNCRKTGHLARDCPNDPICNLCNVSGHVARQCPKANMLGDRGTSGGMRGGGGGYRDVVCR
NCQQLGHMSRDCMGPLMICHNCGGRGHLAYECPSGRFVDRYPRRY

>A.ipaensis-XP016201455, 4 CCHC, cold shock domain-containing protein 3
MAEERYSGVVQWFSNSKGFGFIKPDQGGDDLFVHHSSIQSDGSFRTLVDGDRVEFSIADNSDKPKAL
DVTGPNGAPLRSAPDSARAGNDNRRSAGAGGGGGGGACYQCGDFGHLARDCSRAGNGGGGGGGGXHA
RHMARDCSGGGGGGNGGGCFKCGEFGHMARDCSGGGGNGGGGCYRCGEVGHLARDCNREGGSGAGGN
GGKSTCFNCGKPGHFARECVEASG

>Ca.cajan-XP020223827, 6 CCHC, zinc finger CCHC domain-containing protein 7
MVLRKLLRGPRYFDPPGNSWGACFNCGEEGHAAVNCSAAKRKKPCYVCGGLGHNAKQCTKTQDCFIC
KKGGHRAKDCPEKHASTSKSICICLKCGNSGHDMFFCKSDYSLDDLKEIQCYVCKRLGHLCCVNSDD
ATPGEISCYKCGHLGHTGLACSRLRGEIASGATPSSCFKCGEEGHFARECTSSIKAGKRNYESSNTK
DKRPQKENDYMGNRSAPNDVRHRKKRSSTEERGFSTPKKSKSRGGWMVEHPAEEKDYTSPKKSKHRG
GWTSEHPPEQRGYTTPMKSKSRGGWKTEHPEEFFPPMSTRSSFRSSGTPSSISTRIHSFGNGSHTPS
YKSSKRWNGYTGTPFSQEASARSNHRYSASRFGNSSSDGHGRNYNWW

>Ca.cajan-XP020235098, 5 CCHC, DNA-binding protein HEXBP
MVSQRQRLARKRFKAEHPELFPKPEPTPPKDPDKKKKKKNSAFKRKRPDSKPGSRKRLLRVPGMKPG
DTCFICKAVDHIAKLCPQKAEWEKNKICLRCRRRGHRAKNCPAVQDAANDGKYCYNCGETGHSLSQC
PHPLEQGGTKFAECFVCNQRGHLSKNCPQNTHGIYPKGGSCKICGGVTHLARDCPDKGKKGFVAANG
PFDGSMRTEVRPCGQVTKFISGDDIEDDFMADDMHSADKKKSAKSTDGHVKPKKKGPKVVNFN

>Ca.cajan-XP020218727, 9 CCHC, zinc finger protein GIS2
MSSDSRSRSRSRSRSRSPMDRKIRSDRFSYREAPYRRDSRRGFSRDNLCKNCKRPGHYARECPNVAICH
NCGLPGHIASECTTKSLCWNCKEPGHMASNCPNEGICHTCGKAGHRARECTAPPMPPGDLRLCNNCY
KQGHIAAECTNEKACNNCRKTGHLARDCPNDPICNLCNVSGHVARQCPKANVLGDRSGGGGGGGGG
GGARGGSYRDVICRNCQQLGHMSRDCMGPLMICHNCGGRGHLAYECPSGRFMDRYPRRY

>C.arietinum-1-A1-Ca10268=XP004491141
MGTKKKSAKKIELEEDAADASITLISDDDDEANKDLSLEIVQKALLSRATNPQNDAVLNVVEVEEEE
KEDVEKIKAPEKEQHGEAETGMVDPSDNVVLRVTGSDSISEEIKTHKKKKKKKKPESEIQTVNAAIV
ENEEEKESVETIEVPEKVGPVEAEIGVVDTNDNVVLRKLLRGPRYFDPPSDSVWGTCFNCGEEGHAS
FNCTVAKRKKPCFVCGSLIHNVKKCTMARYCSKCKIVGHRTKDCDQEKHTWGSNSKSLTICLRCGNS
GHDMFLCKNNYSQDDLKVGNRKHKLSSTETPRFQKENSYMGYRSAPHESGEASKKIRPHEEESDIKT
PKKSKYKGGWMTEHPGEFSTSNSKREIWRSPVSPHTKNHFFNNGSLSSKSFKMRNDHDYDGTPNSGG
SARSFHHRYSASRFGNASSDGFERIYNRW

>C.arietinum-2-A2-Ca07571=XP004502023
MMARDHEQVAEINDNLDGASTPSLVFSSDDDDEEANQDLSLKIVEKAMRTREAKLSPNDDVSDEPSG
PAAFEVMEESLNRSVIIAAEQEVEEIIKTTEKDESVEASAVQIGDNAVLRKLLRGPRYFDPPDSSWG
ACYNCGEEGHAAVNCTAAKRMKPCYVCGGLGHGAKQCTKAQSCFICKKGGHRAKDCPEKLMTARVSK
SLTICLKCGNSGHDMFSCKNDYSRDDLKEIQCYLCKTFGHLCCVNTVDAIPGEISCYKCGQMGHTGL
ACSRLQSETTGAASPSLCYRCGEVGHFARECTSSTKAGKKNSEFSNTKKRRSYKENDFRGHWSAPHD
AGKMHKKKRPLPDERGFTTPKKSKSRGGWSRELPTEERGLTTPTKSRSRGGWTMEHPAEERNFKRGF
TTPKKSKSRGGWTMEHPADERDFNSPKKFKSRGGWTAEYAGEFSSSKSKRSSWRSPGTPSARSTKIH
SRSSGSQTPGWSYKSSQGWQGQQPGASNYQGLAMDFHHRYSASRFGNSSSDGYRRSHWQ

>C.arietinum-3-B-Ca04468=XP004496562
MVSQRQRLARKRFKEEHPELFPKVEPTPPKDPLKKKTKNKFKRKKPDSKDKPRSGKRPLRVPGMKPG
DTCFICKGIDHIAKFCTQKAEWEKNKICLRCRRRGHRAQNCPEVHDGSKDVKYCYNCGDTGHSLANC
HQPLQEGGTMFAQCFVCNQQGHLSKNCPQNAHGIYPKGGCCKICGGVTHLARDCPDKGQNGSGAAKG
PVHNLLRTNERPIGQVTKFVSGDDMEDDFTAAQIKDDKSKPSKLKDDNVKPKKGPKVVNFD

>C.arietinum-4-C1-Ca11100=XP004505705
MEGSGKAKATATATGCYKCGKPGHWSRDCPFSLPNSNPNTNTNADPPPPPTSSFKPRSAIDKPKKLP
RTRPKLTPDLLLSDDGLGYVLRYFPRNFKYHGRGHEVRDLGNLIDLYSDWHSRLLPYYPFNQFVHKV
EKVAATKRVKTCLRELRERVANGGDPTKLREPPVVQDIQDDEQGNGEASHLENELFPEPENVNDIQE
DMFNDIFNKATEEPSEPMQNVISASTDPISSAIEKTSNEVPNSGASLSIKAEITAEQRARMEANRLK
ALERRAARANISQSS

>C.arietinum-5-C2-Ca04469=XP004496566
MEDTIDLNENKMLTEEKPETFTSPELKQEDGTIKKKKHTSRKPKKINANVTQPIRNPNPQVCSEPLE
ASPITQLKPNEESTLPDSNKTPMKKKKRRTKKNVLNSQQPQPQPLSQSQPVPEPEPESKGHGKAELN
DRKTNEEQKGEESNETKPSLDIALSTTEIEKKQEAEPIQVYAEGATTPTGKKKKQKKRKKKDEAAVE
NQTNVLHLENAVNHDQIQMNTTDTEHKEERHRSVLDGEQFNEHHVKPLDTAVQRPMELVTEPSIHPI
ESECHIEAPPTDPIILIDPHPATPIEAEQKIVKKSKIEGNAELNDKKKNEEKQGEESNETKPSSDIA
VSTIEIEKKQEAEPIQVSAEGATTPTGKKKKRRKSKRKKRDEAAVAVENQTNLLHLENAVNHDQDQM
NTTDTEHKEKRHRSVLDGEQFNEHNVKPLDTAVQRPMELVTEPSIHPIESECHIEAPPTDPTILIDP
HPATPIDAEQKIVKKSRKRKRKGGLKSDGEAKPNEHNGEPHETLVQKLMGPVTAPSTIQMDPNRETG
FQRPMEPVTEPSTDPINPECHIEEPSTDAIILTDPHPATPIVAEQNIVKKSRKRKRKGGPKSDGEAK
PNEHSVELQETLVQKPIGSVTALSTIPTHHHPETDVQRPMKPVTGPSTDPINLECHIEAPSTDPIIL
MDPHPATLIVAEQNIVKKSRKRKRKGGLKSDGEGKPNEHIGEPRESLVQKPMGPVTAPSTIPTDPHT
SSPIDSEQKKPKKRKKSKKRKGDLKSGDGLPNEVNGLNGKQPEISVQNSIDPIIPAPSVAPTVPIDP
AIPKDPHPATLVKPKQKTSTTESKKKSEKKVKERKKTPMKKPRWKRELEERNAAKELVEVDKLQYHP
LYHLGPNFTTCWACRQPDHTIKECKELRRVAKNAEICFFCGEIGHSLRKCSVSLAGGGRLARCLFCH
AHGHFSYKCPGNSPVPTSEASGMAVNDECKLKFLELKAKRNYRFIVFKIENQEVVVEKLGGPEETYE

DFNASLPSDECRYAVFDFDFTTNENCQKSKIFFIAWSPDISKVRHKMVYASTKDRFKRELDGIQVEL
QATDPSEMSFDIIKARAL

>C.arietinum-6-C3-Ca25010=XP004516521
MSSKKKRKQSDSDEYDDIFFYRYCASSSNNNQAQIQAHVQAQPKSNNRESSVGGTGEPLAPSKSTLY
VSNIDYSLTNSDLHTLFSTFGKIARVTVLKDRHTRLSRGVAFIQFVSRHDAQRAVTEMNKKILNGRT
ITASIAADNGRAPEFIRKRVYNTETALCFECGEHGHLSYECPRNQLGSRSRPQPKKPRRGLNWNKDI
EEEEDEEEEGGDRFDETWASVVDDGACERLLGRNEFR

>C.arietinum-7-C4-Ca10614=XP027193473
MAVNLTQGAIITMCFTSEELQPVLQVIDLKLVQSQQNSSTERYRLVLSDGSYYQQGMLATQKNELVH
SGRLQKGSIVKLHQFICNDVQNRKIIIIVELDVILDKCDLVGEPVPAPKEAPSQSAVSQARNVQSAA
GQSGNTTGISQSLNSNSHAGGINARPNVSMPSMDRPKVNPPASSVYSNSSEPVRYGASNAPPSYPKP
EPGSSLNRPAPSNGSYGVQNTSFRNPQFEASKPMQNSNARPPQPMYRPQSSMYTNTNTNRGPIGRND
APPRIVPISALNPYQSNWTIKARVTAKGELRHYNNARGEGKVFSFDLLDSDGGEIRATCFNAVADQF
YNVIEHGKVFLISRGNLKPAQKNFNHLPNDHEITLDITSVIQPWLEDDNSIPKQIFNFRPIGDIESM
ENNSIVDLIAVVTSISPTASIMRKNGTETQKRTLQLKDMSGRSVELTVWGNFCNVEGQRLQVICDSG
AFPILAVKAARINDFNGKSVGTIGTSQLFVEPDSAEAYTLREWFDKEGRNVPSQSLSRESSSFGKSE
VRKTVSQIKDEKLGTSEKPDWITVCASVSFVKVDNFCYTACPIMIGDRQCNKKVTNNGDGKWRCDRC
DQSVDACDYRYILQLQIQDHTGVTWATAFQEGGEEILGIPAKDLYFLKYEEHDDERFAEIIRKVLFT
KYVFKLKVKEETYGDEQRVKSTVVKAEKVIFASESRFLLDLIDKLKSEKAEGATINSVINNTGLGSF
GQTTPPVYNPIKPNTNTGRDYGMPANQVGQYGNQYSSSVASSAAPGSYVSCSNCGASGHSSAQCLNL
RNQSGQSTGAAYGNRVSAGSGAASGDCYKCHQPGHWARDCPGMGAAAHSDQPMQRPSAGSGAASGEC
YKCHQTGHWARDCPGISAAPPSYGGNNVMQGRYGNGIAQNQQYGGY

>C.arietinum-8-C5-Ca10613=XP027193284
MGAAAHSDQPMQRPSAGSGAASGECYKCHQTGHWARDCPGISAAPPSYGGNNVMQGRYGNGIAQNQQ
YGGY

>C.arietinum-9-D-C04752=XP004500771
MSSDSRSRSRSRSRSPGVRKIRSDRFSFRDAPYRRDSSRGFSRDNLCKNCKRPGHYARECPNVAVCH
NCGLPGHIASECSTKSLCWNCKEPGHMANSCPNEGICHTCGKAGHRARECSAAPMPPGDLRLCNNCY
KQGHIAVECTNEKACNNCRKTGHLARDCPNDPICNLCNVSGHVARQCPKSNVLGDHSGRGGSLRGGG
GGGGGGYRDVVCRNCQQLGHMSRDCMGPLMICHNCGGRGHLAYECPSGRFVDRYPSRRY

>C.arietinum-10-E1-Ca17110=XP004499769
MARVRRKSLQDEEEQEKIRRKGKRKQASNVDDDEEDLDEFEVDGFIVDNVDDEEDGGEEENSKQTQN
KKKKRKRSSKNVLDDDDLELIRENKSLNKEKMSDGKFKRLKKTGVHTEPMEDSSDDEGSLFDDLFED
ESNDAEDDDMSDFIVDEEADVRGKGDSLRQKKSKDMKHSSSLSKEAKRRSGKSHIVSDDPKNMYIDR
EGNSVAHTDIPERMQIIEEAVGSIPVDRMSIEEESSWILRQLASNINPLFSEAKSCGLVDTINGEDI
VRFLELHHIKKYDIPFIAMYRKEQCPSLLEDGKQGDSENTLSDDSESKPKLNWHKILWIIKELDVKW
LHLQKRKSMLQRYYNKHFEEECQMSFLAEESSFRKQIFDSITNMLEKAETEREIDDVDMKFNLYFPP
ADEFLSSGYKRPLMKTYYSDCRKAGLSSVARKFGNPEKFGSLVTLNKVGMDSEEDPEESPEEMASIY
KCETFQTSEAVLKGARHMASLMLSCEVPFRKYVRSIFMDKALVSTNPTMKGNIAIDSFHEFAGFKWL
KDKPLLKFEDFQWLLIQKAEEEELLKVEIKFPEDAIKELMTTCNDAFLKGSEGTSTQLWNEQRKSIL
QDTISNFLLPSMEKEARALLHAKAKNWLLMKYGMQFWNRVSVAPYQNNDNATAQERGVVACCWGNGK
PGTTFVMLDSKGELVDVMHAGSLTLRSQNINDQQRRKSDQMLVHKFLTVHRPKVIVLGAANASCIRL
KEDINEIISMMSEDNFQDVSQEMNGLPAVVLGDEGLPHLYEDSEISTSQLPRQYGIVKRAVALGRYL
LNPLAMVATLCGVNKEVLSWKLNTLERFLSSDEKMEMIEWIMIDITNQVGIDINLAIRHDWLLAPLL

FISGLGPKKAGILHRELLGGTDVRNRKDLAKFGLNTKRVFCNAVGFLQVSCDDPNFVDTAGDILDRT
RIHPESYSLAEELARAVVTKHYADANDTEVNPIECIQNDPKLLESFDMNEYVDSLETTKGEYKRFTL
LDIKMELLHGFKDPRTPYQEPTQDDEFYMVTGETGVALIEGERVQATVRRVLSRQAFCVLESGISGI
LFREDFSDDIGDIPLTEKLREGVVLTCKIKLIDKNRCQVNLTCKVSELKNVGEQSFCDTDPYYCQGN
IILPSQQETTDKKEFVNKNFMSRKISHPHFQNITADQAEEFLEDKAVGEYIFHPSSKGLCYLTLSLK
FFDALYVHKDILEGGKSHEMKSLVELGKTLKVGDEIFENIDEVIGNYVNPLVAHLKDLINFRKFKKG
TKAEVDELLKHEKEEYPNRIPYGLGISYEHPGVFILSYIRSTNPHHEYIAIHPKGFKFRKQIFNNVE
QLMAYFQTHINDNVTKDQSKDYNDSGSGRGRGRGRGRGRGSGACHKCGESGHMSRECTQEGGGGGGR
GGGGSCYKCGESGHMARECTQEGGGGGGRGGGTCYKCGESGHMARECTQEGGGGGGWGGGGSGTCYK
CGESGHMARDCTQESGGGGGRGGGTCYKCGESGHMARDCTQEGGGGGGWRGSSGRGGGRGRGRGRGS
ERSSFSHDDSIDANDSGGFASSKWGVDSNNATNKSFAAGKSWGREGGQNINEGSGNENSGWSAGKNA
TPSSGGSGWGGTGGKSWGGNNTNEQSNIEKGGWGLAAASNSESGNENSGWSSAPVKNETPSGGENGW
GATGGKVWGGDSTNKESNTTKGGWGVTAASSGASGNENSGWSAPDGKNATPSGGESGWGGTGGKSWG
GNSTNEESNTTKGGWGVTAASNGASGNENSGWSASHGKNATPSDGESGWGGTGGKSWGGNSTNEESN
TSKGGWGVTAGSNGGSGNENSGWGAAHGKNATPSGGESKWGGTSSGSGWGGSGGKSWGGSSTNEESN
IAESGGSGYGGGGGRGGGGACYKCGESGHMARECTQEGGGGGGRGGGGRGGRGGGGACYKCGESGHM
ARDCSQGSSEGGGWGGGGRGGGRGGGACYKCGESGHMARECTQEGGGGGGRGGGGACYKCGESGHMA
RECTQESGGGGRWGGGGGGRGGGGGRGGGACYKCGESGHMARDCNQEGGGGAGRYGSGGSGGNCYKC
GESGHFARECPASTG

>C.arietinum-11-E2-Ca09965=XP004509364
MRGGNNGGGIGGGIGGTSCYRCGGIGHIARDCATARVGSGGGGCYKCGEVGHIARDCSIEGGRFEGG
RFGGNNNSGGGGNGGKNTCFNCGKPGHFARECVEASG

>C.arietinum-12-E3-Ca11038=NP001296623
MSSRLTGKVKWFNDQKGFGFITPDDGSEELFVHQSQIRSDGFRSLAEGESVEFEIASDNDGRSKAVE
VTGPDGANVQGTRGGGGGGRGGYGGGGGGYGGGGYSGGGGGGYGGSRSGGYGSGGGGYGGGGGYGGG
RGGGRGGGGGGACYNCGESGHMARDCSQGGSGGGGGGRYSGGGGGGGAGGGSCYSCGESGHFARDCP
TSAR

>C.arietinum-13-E4-Ca05223=XP004504758
MSDEDEYRCFIGGLAWSTSDRKLKDTFEKFGKLTEAKVVVDKFSGRSRGFGFVTFDEKKAMEDAIDA
MNGIDLDGRTITVDRAQPQQGSGKDDGDRYRERGRDRDRDHRGGRGSSDGGCFKCGKPGHFARECPS
EEGRGGRYGGRESRYGGSSGGGGGGYYGPDRNADRSSGGRSRDSGHRGDSGSDRYNRDRAGPYERER
RGSGGFR

>C.arietinum-14-E5-Ca01847=XP004501230
MTRVYIGNLETGISRRELEKDFLVFGAIRNVWISRKPPGYAFIDFYNSGNAEDAIRELDGRHGWKVE
LSFTSGGRGGGGVGREGGGGVGRGGGGGGEGRGGGGGWFGGEGSKLNCFLCGEPGHFAQECPGGDSG
RRRSRSPPRRINSPRQRSLSPRRRSYSKSPPDHERDSHEGVKHANGNGLKDARRSRS

>C.arietinum-15-E6-Ca10996=XP004498562
MSRVYVGNLDSRVTERDLEDEFRVFGVIRSVWVARRPPGYAFIDFDDRRDAQDAIRELDGKNGWRVE
LSHNSRSGGGGGGGGGGRGRGGGGGGGSDLKCYECGEPGHFARECRNRGGSGRRRSRSPPRFRRSPS
YGRRSYSPRGRSPRRRSVSPRGRSYSRSPAPYRGREEVPYANGNGIRERRRSRS

>C.arietinum-16-E7-Ca14507=XP004509838
MARVYIGNLDPRVTERELEDEFRLYGVLRSVWVARRPPGYAFIEFDDRRDALDAIHALDGKNGWRVE
LSHNSKDGGGRGGGRGGRGSSDLKCYECGEPGHFARECREPRGSRGLGSGRRRSPSPYYRHRRSPSY
GSGRRSYSPRGRRSPRRLSITPPRGRSYSRSPPYRYSRRDSPYANGYD

>C.arietinum-17-E8-Ca11994=XP004493916
MTNDPDIIKRKKISTMPRYDDKYGNTRLYVGRLSSRTRSRDLERVFSRYGRVRDVDMKHDYAFVEFS
DPRDADDARYNLDGRDVDGSRLIVEFAKGVPRGSRDSRDSREYLGRGPPPGSGRCFNCGIDGHWARD
CKAGDWKNKCYRCGERGHIEKNCKNSPKKLSRHARSVSRSPGRSRSPRRGRSRDRSYSPARSYSRSR
SPVRRDRSPIPEDRSRSPQPSKSRKYSRSPEGSPQKSTSPGNDRVVATQDGSDYSDGPRVKSRSPSR
DNDDSPKANGRSRSRSRSRSRSPRDDRSPIEEDDDNRRHSPSP

>C.arietinum-18-E9-Ca05466=XP004497121
MMERDTGRPRGFGFITFADRRGMEDAIKEMDGREIGDRIISVNKAQPRMGGDDADQGYRGGYSSGGR
GSYGAGDRVGQDDCFKCGRPGHWARDCPLAGGGGGRGRGGGSFSSRPRFGGAGGHGDRLGERYIDDR
NDGGRYADRDRLDSRDYKYSGRDRYAGDRYTTSGDRFASDRYGSGSDYPQNGYGKERGYDRYSGARG
GADRYGSGMPARDEGRNYRGRAGPYDRPSRTARPSSFDRY

>C.arietinum-19-E10-Ca13113=XP004498355
MSVLIVTSLGDLVIDLHTQKCPLTCKNFLKLCKIKYYNGCLFHTVQKDFTAQTGDPTGTAAGGDSIY
KYLYGDQARFFNDEIHIDLKHSKTGTVAMASAGENMNASQFYITLRDDLDYLDGKHTVFGEVAEGFE
TLTRINEAYADEKGRPFKNIRIKHTYILEDPYDDPPQLPEFIPEASPEGKPKDEVDDEVRLEDDWVP
MDEQLNPGELEEVIRSKEAHSRAVVLESIGDIPDAEVKPPDNVLFVCKLNPVTEDEDLHTIFSRFGT
VSSAEIIRDHKTGDSLCYAFIEFDDKQACEQAYFKMDNALIDDRRIHVDFSQSVAKLWSQYMRKDNK
GGGCFKCGSTDHIARDCTGDAIMKQPTKFILKDNNAQRGGDKASYEMVFDGDNTESPRRQTKHQRHD
RDAQDDKNGRKENFKNDSHRGRRDQGMAGSNSRERYGDRSRGLEENGDDKTRLERGARDSDSHVDKK
DRERHMGRHRDDEYRRKDELDTRKRDLDSSYTERRASRDDSIKTDASHLDRRNDRDYRKRTEDSGRQ
NVKIDSGRRKRSPDDEDYKRRRNDEDYREDVKIDSGRRKRSPDNGDYRHRKDDEDYKRRKEDRGHRR
HDTESDDYHRRHHGDRR

>C.arietinum-20-F1-Ca24838=CAC44110
MLLGDAEYWWRSTRSLMRSAHEEVNWESFKGKFFDKYFPMSTRTRLGDDFLKLHQGNMTVGEYAAKF
ESLSRHFRFFREEVDEPFLCHRFQDGLKYEIQDFVLPLGIQRFQVLVEKCREVEDMKNKRASRGGNF
NSGGPSRAKNQNKGKQVAKSYNRPHDNNGGQDNPGNQDFGRQVGQGIRCFRCGEEGHYAYACTFNST
TCYNCHKPGHFARNCEAPKAEPMVDVARITLPTARGQVYCVGTDLSSDLIQYDCAITGNLLTTLSDS
EATHSFIAMDYVNCLKFQCLLYC

>C.arietinum-21-F2-Ca26496=XP004514782
MTVGEYAAKFESLSRHFRFFREEVDEPFLCHRFQDGLKYEIQDFVLPLGIQRFQVLVEKCREVEDMK
NKRASRGGNFNSGGPSRAKYQNKGKQVAKSYNRPHDNNGGQDNPGNQDFGRQVGQGIRCFRCGEKGH
YAHACTFNSTTCYNCHKPGHFARNCKAPKVEPMVNVARITLPTARGQVYCAGAEVGNLSSDLIQYDC
AITGNPLTTLSDSGATHSFIAMDYVYRLKLSVSTLLLRL

>G.max-XP006605629, 6 CCHC, zinc finger CCHC domain-containing protein 7 isoform X1
MGRKEKQNAKAIDEEHDVVNFNGASTPSLVFSSDDDEANQDLSLKIVEKAMRMRAAKHAPNDDVSSP
FSQKSELAVPLNDVVSDLPSAIADSEVTEKKKTAKLKREAAGDQSVVIAEEQEMEETSNATENHEFV
EGSPVLIGHNMVLRKLLRGPRYFDPPDSSWGACFNCGEDGHAAVNCSAAKRKKPCYVCGGLGHNARQ
CTKAQDCFICKKGGHRAKDCLEKHTSRSKSVAICLKCGNSGHDMFSCRNDYSPDDLKEIQCYVCKRV

GHLCCVNTDDATPGEISCYKCGQLGHTGLACSRLRDEITSGATPSSCFKCGEEGHFARECTSSIKSG
KRNWESSHTKDKRSQKENDYMGNRSASNDMVGARRKKRSPTEERGFSTPKKSKSRGGWTAEYPTEER
GFTTPKKSKSRGGWTTEHPTEERGFTTPKKSKNRGGWTSEHPLEQKDYTTPKKSKSRGGWMSEHPEE
FFPPMSSRSNGYRSLGTPSSRNNKIHSFGGGSHTPSYKSSKVWNDHAGTSMSQGSARSNHHRFSASR
FGNSSSDGHGRNYNWW

>G.max-XP003542327, 6 CCHC, protein AIR1 isoform X1
MGRKEKQNTKAIEEERDQDNFNGASTPPLVFSSDDDEANQDLSLKIIKKAMRMRTAKHAPNDDVSSP
FSQKPDLALPPSGGVSDGPSAIADSEVMEKKKTAKLKVEAGDQSVVIAEEQEMEETINATENHVEGR
PEIGDNMVLRKLLRGPRYFDPPDNSWGACFNCGEEGHAAVNCSAVKRKKPCYVCGCLGHNARQCSKV
QDCFICKKGGHRAKDCPEKHTSTSKSIAICLKCGNSGHDIFSCRNDYSQDDLKEIQCYVCKRLGHLC
CVNTDDATAGEISCYKCGQLGHMGLACLRLQDEIASGATPSSCFKCGEEGHFARECTSSINFPPQSG
KGNWESSRTKDKRSQKENRYMGNRSAPNDISGARRKKRSPTEERGFSTPKKSKSRGGWMAEYPTKER
GFTTPKKSKSRGGCTTEHPSEQQDYATPKKSKSRGGWTSEHPEEFFPPLSSRSKGYRSPGTPSSRND
RIHSFGGGSHTPSYKSSKVWNGHTGTSMSQGSARSNHHRYSASRFGNSSSDGHGRNYNWW

>G.max-XP003522851, 9 CCHC, zinc finger protein GIS2
MSSDSRSRSRSRSRSPMDRKIRSDRFSYRDAPYRRDSRRGFSRDNLCKNCKRPGHYARECPNVAICHNC
GLPGHIASECTTKSLCWNCKEPGHMASSCPNEGICHTCGKAGHRARECSAPPMPPGDLRLCNNCYKQ
GHIAAECTNEKACNNCRKTGHLARDCPNDPICNLCNVSGHVARQCPKANVLGDRSGGGGGARGGGGG
GYRDVVCRNCQQLGHMSRDCMGPLMICHNCGGRGHLAYECPSGRFMDRYPRRY

>G.max-XP003527126, 9 CCHC, zinc finger protein GIS2
MSSDSRSRSRSRSRSRSPMDRKIRSDRFSYRDAPYRRDSRRGFSRDNLCKNCKRPGHYARECPNVAICH
NCGLPGHIASECTTKSLCWNCKEPGHMASSCPNEGICHTCGKAGHRARECSAPPMPPGDLRLCNNCY
KQGHIAAECTNEKACNNCRKTGHLARDCPNDPICNLCNVSGHVARQCPKANVLGDRSGGGGGGGGAR
GGGGGGYRDVVCRNCQQLGHMSRDCMGPLMICHNCGGRGHLAYECPSGRFMDRYPRRY

>G.soja-XP028222014, 6 CCHC, zinc finger CCHC domain-containing protein 7-like
MGRKEKQNAKAIDEEHDVVNFNGASTPSLVFSSDDDEANQDLSLKIVEKAMRMRAAKHAPNDDVSSP
FSQKSELAVPLNDVVSDLPSAIADSEVTEKKKTAKLKREAAGDQSVVIAEEQEMEETSNATENHEFV
EGSPVLIGDNMVLRKLLRGPRYFDPPDSSWGACFNCGEDGHAAVNCSAAKRKKPCYVCGGLGHNARQ
CTKAQDCFICKKGGHRAKDCLEKHTSRSKSVAICLKCGNSGHDMFSCRNDYSPDDLKEIQCYVCKRV
GHLCCVNTDDATPGEISCYKCGQLGHTGLACSRLRDEITSGATPSSCFKCGEEGHFARECTSSIKSG
KRNWESSHTKDKRSQKENDYMGNRSASNDMVGARRKKRSPTEERGFSTPKKSKSRGGWTAEYPTEER
GFTTPKKSKSRGGWTTEHPTEERGFTTPKKSKNRGGWTSEHPLEQKDYTTPKKSKSRGGWMSEHPEE
FFPPMSSRSNGYRSLGTPSSRNNKIHSFGGGSHTPSYKSSKVWNDHAGTSMSQGSARSNHHRFSASR
FGNSSSDGHGRNYNWW

>G.soja-XP028197625, 6 CCHC, protein AIR1-like isoform X1
MGRKEKQNTKAIEEERDQDNFNGASTPPLVFSSDDDEANQDLSLKIIKKAMRMRTAKHAPNDDVSSP
FSQKPDLALPPSGGVSDGPSAIADSEVMEKKKTAKLKVEAGDQSVVIAEEQEMEETINATENHVEGR
PEIGDNMVLRKLLRGPRYFDPPDNSWGACFNCGEEGHAAVNCSAVKRKKPCYVCGCLGHNARQCSKV
QDCFICKKGGHRAKDCPEKHTSTSKSIAICLKCGNSGHDIFSCRNDYSQDDLKEIQCYVCKRLGHLC
CVNTDDATAGEISCYKCGQLGHMGLACLRLQDEIASGATPSSCFKCGEEGHFARECTSSINFPPQSG
KGNWESSRTKDKRSQKENRYMGNRSAPNDISGARRKKRSPTEERGFSTPKKSKSRGGWMAEYPTKER

GFTTPKKSKSRGGCTTEHPSEQQDYATPKKSKSRGGWTSEHPEEFFPPLSSRSKGYRSPGTPSSRND
RIHSFGGGSHTPSYKSSKVWNGHTGTSMSQGSARSNHHRYSASRFGNSSSDGHGRNYNWW

>G.soja-XP028227280, 9 CCHC, zinc finger protein GIS2-like
MSSDSRSRSRSRSPMDRKIRSDRFSYRDAPYRRDSRRGFSRDNLCKNCKRPGHYARECPNVAICHNC
GLPGHIASECTTKSLCWNCKEPGHMASSCPNEGICHTCGKAGHRARECSAPPMPPGDLRLCNNCYKQ
GHIAAECTNEKACNNCRKTGHLARDCPNDPICNLCNVSGHVARQCPKANVLGDRSGGGGGARGGGGG
GYRDVVCRNCQQLGHMSRDCMGPLMICHNCGGRGHLAYECPSGRFMDRYPRRY

>G.soja-XP028234529, 9 CCHC, zinc finger protein GIS2-like
MSSDSRSRSRSRSRSPMDRKIRSDRFSYRDAPYRRDSRRGFSRDNLCKNCKRPGHYARECPNVAICH
NCGLPGHIASECTTKSLCWNCKEPGHMASSCPNEGICHTCGKAGHRARECSAPPMPPGDLRLCNNCY
KQGHIAAECTNEKACNNCRKTGHLARDCPNDPICNLCNVSGHVARQCPKANVLGDRSGGGGGGGGAR
GGGGGGYRDVVCRNCQQLGHMSRDCMGPLMICHNCGGRGHLAYECPSGRFMDRYPRRY

>Lo.japonicus-Lj6g3v1038840, 6 CCHC, zinc finger CCHC domain-containing protein 7-like isoform X1
MGRKERPKAKVAEPEANGDLNGGSTPPFVFSSDDDEANQDLSLKIVEKAIRMRTAKLASNDTVSNGG
GGGGGVVLPSQQSELAAALSDGVLDWPSVIAGSQVKEKKKKKKTVLTVESGDQGVVRAEEQEIEGTI
EAAENQEAVEASMVQVGDNMVLRKLLRGPRYFDPPDSSWGACYNCGEEGHAAVNCTAAKRKKPCYVC
GGLGHSAKQCIKGHDCYICKKGGHRAKDCPEKYTRASKSLTVCLRCGNSGHDMFSCKNDYSLDDLKE
IRCYICKTFGHLCCVNTENATRGEVSCYKCGQLGHTGLACTRLRGESTGASMPSSCFRCGEEGHFAR
ECTNLIKAGKKNSEFSNTKTKNSYGENDYMGHRSAPPDLGKTRNKRRPPTEDRVFKTPKKSKSRGGW
TTEYPAEERGFTTPKKSKSRGGWMTEHPAEERDYTTPKKSKNRGGWMTEHPGEFSPSSSQRSNQTNA
WTASTRSTQFYAHGSGSHTPSTNYSSRVWQGQPGASNYQGPDYTFHRFSASRFGNSSSDGHWRSYDR
YATNKNTPVSKLKIKEESLLISNSFF

>Lo.japonicus-Lj2g3v2905060, 4 CCHC, Cellular nucleic acid binding protein
MEHTEAGTVQAVLFQEEAVETIKGTQKMEPSEAGTVQMSDNVVLRKLLRGPRYYDPPADCGWETCYN
CGEEGHATVKCAAAKELKKPCYLCGSLMHQAKRCKKERDCIICRKVGHPVKNCRRTHMGDSICLRCR
ISGHDMFSCGNFYSMYDLKEIQCYVCKSFGHLCCANTTGSTPIEISCYKCGQTGHTGLVSFLFKELS
YMVRYESHE

>Lo.japonicus-Lj4g3v1037770, 7 CCHC, Cellular nucleic acid binding protein
MAEERFSGVVQWFNNVKGFGFIKPDDGGEDLFVHQSSIRSDGYRTLLEGDLVEFSIATGDNDKTKAV
DVTGPNGAALQPTRKDSAPRGFGGWRGGERRNGGGGGGGGGGCYNCGDTGHLARDCHRSNNNGGGGG
GAACYNCGDAGHLARDCNRSNNNSGGGGAGCYNCGDTGHLARDCNRSNNSGGGGGGGACYTCGGFG
HLARDCMRGGNGGGGGPGSASCFRCGGIGHMARDCATAKGPSSGGVGGGGCFRCGEVGHLARDCDGG
VAVSGGGGNAGRNTCFNCGKPGHFARECIEASG

>Lo.japonicus-Lj5g3v2013630, 5 CCHC, zinc finger CCHC domain-containing protein 7-like isoform X2
MVSQRQRLARKRFKAENPELFPKPEPTPPKDPDKKKKKKRKTTPFKRTKDDSKGVKSNSRKHPLRVP
GMRPGETCFICKARDHIAKLCPEKAEWEKNKICLRCRRRGHRAQNCPEVKDGPKDGKFCYNCGETGH
PLSNCPQPLQEGGTKFAECFVCKQRGHLSKNCPQNAHGIYPKGGSCKICGGVTHLAKDCPDKGNRGS
VAANRPLRISSRTEERPSSHTRFVSGDDMEDDFMADDINSGDKNTSSNSKDGHVKPKKGPKVVNF

>Lu.angustifolius-XP019455875, 6 CCHC, protein AIR2-like
MGRKERQKKKKTHSSDDDAEANEDLSLKIVQKALAKREHKPNQLPNDVVVDGDVGAKRNEVVEVRND
DVLVGKSVIGVSDVGNEETRVEKKKRRKKKKVEYEDQSVVIAEEQGAQEVITATEKNECAEVDKTDQ
TSDNIVLRKLLRGPRYFDPPDSSWGTCYNCGEEGHAAVNCTAASRRKKPCFVCGSLEHNVKQCTKSR
DCFICKKGGHRAKDCPERHNAVGASISSICLKCGISGHEMFSCKDDYSKDDLKEIQCYVCKRFGHLC
CVDNTDTAQRQFSCYKCGQLGHTGLACSRLRSETTDASTPSSCYKCGEEGHFARECQSSVKPKKYGL
SNTKSSRFQKENDYMRYRSAPYDMDKSYKKKRHHTEEREDTTPQKSIHRGDWTTEHPGDFSPVKSKR
NGWMSPVTPSTESTKLHSFSNGSHSPSSKSYKARNAYETPGSRRSGKAFHHRFSASRFDNSSGDGSG
RNYNWW

>Lu.angustifolius-XP019433543, 9 CCHC, zinc finger CCHC domain-containing protein 7-like isoform X1
MGSQDNQKANVADHKNDIELNDDAAFIPLVVLSSDGDDEEVNPDRNFVAPKADVLNGVVSITSSQQP
EPVTENDCVLDEMKKTESGDQSVIIVPEEQETVKTIEATENVQLGNNIVLRKLLRGPRYFDPPDSGR
GSCFNCGVEGHVALNCTEERRKKPCYVCGGLGHNAKQCTNNVSTGKNCFLCNKGGHRAKNCPEKHGN
ASEVLRICLKCGNSGHDMFSCKNDYRLDDLEEIRCYICKRFGHLCCANTDETRPREISCYKCGRLGH
TGLACSRFRVEATGAATAGSCYKCGGEGHFSRECTSSMKASPRFQVETTGAATPGMCFKCGQEGHFA
RECIFQASPRFRVETTGAATPVSCYKCGQEGHFARECIFQASPRFRAETTGEATPGSCYKCGQEGHF
ARECSFQARKRVHGLSNTKTKRSHTEDDYMGYMSAPHQMGKTRKKKPTLMDERGFTTPKKSKNRGGW
ISDHYAEESEFTAQKKSKNKRGWMTDHYTEEGFSTPKKRKSRGGWMTEHPADFSPSKSGKSSWKSTG
TPSIRSNNIYSHGSVSHTPRSKSSNRWNSHGGTSKSHGSKAPHHRFSASRW

>Lu.angustifolius-XP019452795, 5 CCHC, DNA-binding protein HEXBP
MVSERQRVARKRFKSEHPELFPKPEPTPPKDTEKKKKKKKKKKKNNTPEQLGVTTKPFKSNSRKHP
LRVPGMKPGESCFICKAKDHIAKSCPEKALWEKNKICLRCRRRGHRATNCPELQHGTNDDKYCYNCG
ENGHSLAYCPYPLQQGGTKFAECFVCKQRGHLSKDCPQNAHGIYPKGGCCKICGGVTHLAKDCPDKG
RQGSVAANGPYNRLIGAGVRATGQVTKFVSGDDIDDDFVTDDINSGDKNNSSKSNEGDVKPKKGPKV
VVFN

>Lu.angustifolius-XP019438188, 9 CCHC, zinc finger protein GIS2
MSSDSRSRSRSRSRSPMDRKIRSDRFSHREAPYRRDSHRGFSRDNLCKNCKRPGHYARECPNVAICH
NCGLPGHIASECSTKSLCWNCKEPGHMASNCPNEGICHTCGKVGHRARECSAPPMPPGDLRLCNNCY
KQGHIAVECTNEKACNNCRKTGHLARDCPNDPICNLCNVSGHVARQCPKANDLGERFRGGGGGGGIR
GGGGGGYRDRDVVCRNCQQLGHMSRDCMGPLMICHNCGGRGHLAYECPSGRMMDRYPRRY

>M.truncatula-ABE91952, 1 CCHC, zinc finger, CCHC-type
MNSDFVFSAHDPTNLHQSKKPPDTIKQSSQKPSFRDKLLESNQDIPIREKENMIEKKLVRIELEEGN
RLLPKIYIEPQTFQELCTPWKDALVVKLLGKSLGYNTMKDRLQKIWKLQGGFDIMDNDNGFFMVKFD
QAADKEKVITGGPWLIFDHCLAVTHWTPEFASPNAKVDRTVVWVRFPGLNLVYYDESFLLAMASALG
RPIKVDTNTLKVERGKFARVCVEIDLTVPVVGKIWVNGHWYKVQYEGLHLICTNCGCYGHLGRNCME
KPSTFDPRSPNHHTAGGNNPPSNPEPSQPRQNPTQTCPDAINSQLMACNQNGNSIIDHNKDVTANKR
NAINRIDDNQVLHGDWLVVTRRKKTPIQHSLNASKSVTHKTNRFQALSTMTHHDKSAPINNKFPSWS
KSQEIPRANNRSTETKRRRQEDFYEPIAKNLSTNDSRHITILEPVLSLKTKDTSTSKIDITQPHSTE
HVTNPSQNNHEPVTTYTEDDTIPDPSSSMPQDTPM

>M.truncatula-KEH19794, 4 CCHC, zinc knuckle CCHC-type family protein
MNSCQLVIHMFHTPIRSLTFPCTTKPPHLNIHLLLSLPAPTLLSSSLNDDSFSIPKSSQFGYSSPED
LFGLEVEFKPRKANSHTREPRSWFGPNGQYIRELPCPSCRGRGYTPCAECGIERSRSDCPKCNGKGL
LTCHQCSGDCVIWEESIDERPWERAQSISPLKVKDDDEVDKLDIKLDAKKKSKRVYQSPSPEVGLKI
SRSLKSLNAKTGLFSKRMKIIHQDPTLQAQRVAAIKKAKRTVSARNHASKTMKDFFSDPINREKRSM
AMQGVKFYCQNCGREGHRRHYCPELKDGLIDRRFTCRLCGEKGHNRRTCSKLRISHSDGRVIKHHRC
KICRQYGHNRRTCPQVISNKRMDMTSQRVYKCRLCQKEGHNSRTCPSRIVGIEHSLE

>P.vulgaris-XP007146032, 6 CCHC, hypothetical protein PHAVU_006G006900g
MGRKEKAKAKAIEENGDNHFGGASTPSLVFSSDDDDANQDLSLKIVEKAMRIRAAKRAAPNDNVSSS
QTLELSVARNVGVLDVPSAIADSEVTEKKKTTKLKIETGDQRVVIANEHETEEIIKDTENHESVEGG
AVQLGDNMVLRKLLRGPRYFDPPNSSWGACFNCGEEGHAAVNCSVAKRKKPCYVCGVLGHNAKQCTK
TQDCFICKQGGHRARDCPEKHTSTPRSIAICLKCGNSGHDMFGCKNDYSLDDLEEIQCYVCKRLGHL
CCVNSDDATPGEISCYKCGRLGHTGLACSRLQDEIASGATPSSCFKCGEEGHFARECTSAVKTGKRN
RDSSRTKDKRPYKENDYIGNRSAPNDMGVARRKKRSPAEERGGFSLPKKSKSRGGWMQEHPAEERGF
TTPKKSKSRGGWTTEHPAEHNGYTTPKKSKSRGGWTTDHPEEFFPPMATRNSYKFSGSPYSRSTKIH
SFGSGSHTPSYKSSKVWTVHQGTPMSQVSAWSNHHRFSTSRFGNSSTGGHGRNYSQWQ

>P.vulgaris-XP007143419, 5 CCHC, hypothetical protein PHAVU_007G070700g
MVSQRQRLARKRFKSENPELFPKAEPTPPKDPNKKKKKKKSSAFKRKAESKPGSRNRHPLRVPGMK
PGESCFICRAKDHIAKLCPEKTEWEKNKICLRCRRRGHRAKNCPEVQDAAKDVKYCYNCGETGHSLS
LCPHPLQEGGTQFAECFVCNQQGHLSKNCPQNTHGIYPKGGCCKICGGVTHLAKDCPDKGRQGSVAA
NGPFGRSMRIEERPRGKVTKFISGDDIDDDFMADDTHSADNNKSANSKDGNVKPKKKEPKVVIFN

>P.vulgaris-XP007135925, 9 CCHC, hypothetical protein PHAVU_009G003600g
MSSDSRSRSRSRSRSRSPMDRKIRSDRFSYRDAPYRRDSRRGFSRDNLCKNCKRPGHYARECPNVAICH
NCGLPGHIASECTTKSLCWNCKEPGHMASNCPNEGICHTCGKAGHRARECTAPPMPPGDLRLCNNCY
KQGHIAAECTNDKACNNCRKTGHLARDCPNDPICNLCNVSGHVARQCPKANVLGDRSGGGGGGGGGG
GARGGGGGGYRDVICRNCQQLGHMSRDCMGPLMICHNCGGRGHLAYECPSGRFMDRYPRRY

>V.angularis-XP017437156, 6 CCHC, zinc finger CCHC domain-containing protein 7-like
MGRKEKSKSKTMVELDDDHFTSVSTPSLVFSSDDDEANQDLSLKIVEKAMRMRTAKCAAPSDDVLLS
QTLELAVARNVDVPDVPSAIADSEGKEKKKTTKLKTETRDERVVIANEQEMEETIKDTENQESAEGG
AVQTGNNMVLRKLLRGPRYFDPPNNSWGACFNCGEEGHAAVNCSVAKRKKPCYVCGVLGHNAKQCTK
TQDCFICKKGGHRARDCPEKHASTPRIIAICLKCGNSGHDMFGCKNDYSLDDLQEIQCYVCKRLGHL
CCVNSDDATPGEISCYKCGRLGHTGLACSRLQDEIASGATPSSCFKCGEEGHFARECTSAVKTGKRS
RDSSRTKDKRFHKENDYIGNRSAPNDMGVARRKKRLPTEERGGFSLPKKSKSRGGWMQEHPAEERGF
TTPKKSKGRGGWTTEHPAEHKGYTTPKKSKSRDGWTTEHPEEFFPPMAMRNSYRFSGSPYSRSTAIH
SFGSGSHTPGYKSSKVWTDHDGTPMSQGPAWLNHHRFSASRFGNSSGGGYGRNYSRW

>V.angularis-XP017413280, 5 CCHC, uncharacterized protein C683.02c-like
MVNQRQRLARKRFKAEHPELFPKVEPTPPKDPNKKKKKSSAFKRKRAESKSGSGKRHPLRVPGMKPG
DSCFICMAKDHIAKLCPQKTEWEKNKICLRCRRRGHRAKNCPEVQDAAKDVKHCYNCGETGHSLSLC

PYPLEEGGTKFAECFVCNQQGHLSRNCPQNTHGIYPKGGCCKICGGVTHLAKDCPDKRWQGSVAANG
PGGRSKRIEESPRGQVTKFISGDDIEDDFMIDDVRSGDNNKSANSNDVNVTAKKKDPKVVIFN

>V.angularis-XP017422002, 9 CCHC, zinc finger protein GIS2
MSSDSRSRSRSRSRSPMDRKIRSDRFSYRDAPYRRDSRRGFSRDNLCKNCKRPGHYARECPNVAICH
NCGLPGHIASECTTKSLCWNCKEPGHMASNCPNEGICHTCGKAGHRARECTAPPMPPGDLRLCNNCY
KQGHIAAECTNEKACNNCRKTGHLARDCPNDPICNLCNVSGHVARQCPKANVIGDRSGGGGGGGARG
GGGGGGYRDVICRNCQQLGHMSRDCMGPLMICHNCGGRGHLAYECPSGRFMDRYPRRY

>V.radiata-XP014516861, 6 CCHC, zinc finger CCHC domain-containing protein 7
MGRKEKSKSKAVVELHDDQFTGASTPSLVFSSDEDEANQDLSLKIVEKAMRMREAKCATPSDHVSLS
QTLEFAVARNVVPDVPSAIADSEVKEKKKTTKLKIDTRDERVEEMEETIKDTENQSAEGGAVQTVDN
MVLRKLLRGPRYFDPPNDSWGACFNCFNCGEEGHAAVNCSVAKRKKPCYVCGVLGHNAKQCTKTQDC
FICKKGGHRARDCPEKHASTPRIIAICLKCGNSGHDMFGCKNDYSLDDLQEIQCYVCKRLGHLCCVN
SDDATPGEISCYKCGRLGHTGLACSRLQDEIASGATPSSCFKCGEEGHFARECTSAVKTGKRSRDSS
RTKDKRSHKENDYIGNRSAPNDMGVARRKKRLPTEERGGFSLPKKSKSRGGWMQEHPAEERGFTTPK
KSNGRGGWTTEHPAEHKGYTTPKKSKSRGGWTTEHPEEFFPPMAMRNSYRFSGSPYSRSTTIHSFGS
GSHTPGYKSNKVWTDHDGTPMSQGSAWSNHHRFSASRFGNSSSGGYGRNYSRW

>V.radiata-XP022641333, 5 CCHC, cold shock protein 1
MVSQRQRLARKRFKAEHPELFPKAEPTPPKDPNKKKKKSSAFKRKRAESKSGSGKRQPLRVPGMKPG
DSCFICMAKDHIAKLCPQKTEWEKNKICLRCRRRGHRAKNCPEVQDAAKDVKYCYNCGETGHSLSHC
PYPLEEGGTKFAECFVCNQQGHLSRNCPQNTHGIYPKGGCCKICGGVTHLAKDCPDKGSQGSVAANG
RAGRPMRIEERPRGQVTKFISGDDIEDDFMIDDIRSGDNNKSANSNDVNVTAKKKDPKVVIFN

>V.radiata-XP014501462, 9 CCHC, zinc finger protein GIS2
MSSDSRSRSRSRSRSPMDRKIRSDRFSYRDAPYRRDSRRGFSRDNLCKNCKRPGHYARECPNVAICH
NCGLPGHIASECTTKSLCWNCKEPGHMASNCPNEGICHTCGKAGHRARECTAPPMPPGDLRLCNNCY
KQGHIAAECTNEKACNNCRKTGHLARDCPNDPICNLCNVSGHVARQCPKANVIGDRSSGGGGGARGG
GGGGYRDVICRNCQQLGHMSRDCMGPLMICHNCGGRGHLAYECPSGRFMDRYPRRY

Supplementary material S3. Promoter and CDS regions of two studied genes, *Ca04468* and *Ca07571*, with identified SNP in reference genotypes of chickpea. SNP are shown by different colour corresponding to Figure 2. 'Start' and 'Stop' codons in CDS are indicated by green and red, respectively. Gene *Ca04468* is intronless. In gene *Ca07571*, exons are indicated by yellow and introns are without colour.

Gene *Ca04468*. *Cicer arietinum*, Kabuli, cv. Frontier. Chromosome Ca4

>Cicer.Frontier.gnm1.genomic DNA.Ca4 version 1.0

GCACCACGAGATATTGAGGCATGATATGACTTAAATAAACGCAAAAAAATGAATTTTTAACAAATTT
TGAATCAAAATTATGTTTGTGGATTAAAACTAGGGAGAAACAGAATAGGGGAACTATTTTCGCGATT
CAGCTAAAATAAAGGGATCAAAACTGCAATTAAGCCTATATTTTATTAATATCAATTTTAAGAGATG
AAGAACAATTGAACATATGCATGATACAGAGGCACATGGGTTGAGAATGAGAGAGACGAAATGCAAT
TTCAATTTCTCAAAATATAAACATAAAGGTGTGAGTATATTTTAATAATTTATCACTATTGTTTTGA
TAATCTAATATAATTATATATAAAAAATAATCGATAATATATAGTTATTATATATATATATAAAAGT
TAAAAAATATAGAGGATTGATAATATATTATTATATATATAAAAAATAATCGATAATATACAATTAT
TATATATAAAAGAAAATAAAAAAAATATAAAAGATACCGTAATTTAGGGTTGTGCAAAAAATCCGAT
TATGAGACCCAAATTCAAAATGAGATCCAAATCCATTTTAAATATCCGATTAAAATCATATGGTTAT
AATCTGGTTGGATAACCACTTATGTTTTATATTTGGATTTAGTTTTTATCTACTACCAATATTTTGT
TAATTTTGAGATTATATTTTTTGAAAAAAATTTGAATTTTTCTTTTCTCAAAAAAAATTTAAATCGA
ATTTTTTTAAAAAATTTATCGAAAAAATCAATTTTTTTAAATAACCGATTTTTAAATTATGAATAAA
TATTTTTTAAAAAAATAATCAAATTTTAACCGATTTTAAAAAAAAAATTAATTTTAAAATTAAAATT
TTCAAAATCAGTTGCTTATAAATCGGTTGTTTAACTATGATCAGTTTTATAATTAATTTTATAATCA
ATTTTAAATCAAATAATAAATTTAATTATAAATCTAAATTCATATATTAATTTTAAAATAGGAATTG
TTTGGTTTTTTTTAAAAATCAAACATAAACATATAGCCTCTACCCCCTCCTGAAAAGGTCCGCGCTG
TATACATTTTTTAACCTAAATATTGATAGAAGTTACCTGCAAATATTTTATGACTTAATTATAAAAA
CAATCTAAAGAATTAAATGTATTTTGCCTAAAATAAATAAGAAATAAAAAATAAAAAAAAGGGGCAA
AAGAAAGAAGAAAACCCTGAGGTTTTAGATTGGGGTTATCTCGTCACCGCACCAGCTGCCGCAGCCG
TATCTAACCTAATTAATCAATCAATCATCGGCCACATCTTGTCAGAGATGGTGAGCCAAAGGCAACG
ACTTGCTCGGAAACGATTCAAAGAAGAACACCCCGAACTATTCCCCAAAGTTGAACCCACTCCTCCT
AAAGACCCACTCAAAAAGAAGACCAAGAACAAATTCAAGCGCAAAAAGCCAGACTCAAAAGACAAAC
CCCGTTCAGGAAAACGCCCCCTTCGTGTTCCCGGTATGAAACCCGGTGACACGTGTTTCATTTGCAA
GGGCATAGATCATATCGCAAAATTCTGTACTCAAAAAGCCGAATGGGAAAAGAATAAGATATGTTTG
CGGTGTCGACGACGCGGTCATAGAGCTCAGAATTGTCCTGAGGTTCATGATGGTTCTAAGGATGTTA
AGTATTGTTATAATTGTGGTGATACTGGTCATTCTCTTGCTAATTGTCATCAACCTCTTCAAGAAGG
AGGGACAATGTTTGCTCAGTGTTTTGTTTGTAATCAACAAGGGCATTTGAGTAAAAATTGTCCTCAA
AATGCTCATGGCATTTATCCTAAGGTATATTATCTTATATCATTTCAATTATACTATACAATTTTCT
CTCCAAAGGATAACCATATAAGTTAGTTCAATTAACAACACTTGTTCTTTAAACAAACGGTTTGGCT
CAAGTGATTAATGAGCTCTAGAGAATGACGGAGTGATCGGGATTATCACATTCGACCTCTAGTTGAA
ACAATTGTTGACGAGACTTTACTTATCTTCCGAATGAACTTTTAGATTATCAGTGTCCATTTCCCTT
GAAAATCCGAGTGTTAGAGAAAAAGAACAATAGTTGTTATTTGCAAGTAAGCTGTGTCTATATGCAT
ACCTATTTATTTATTTGTTTTGTGTGTTTGTTTGTAATAGGGTGGTTGTTGTAAAATCTGTGGTGGT
GTGACACATCTGGCAAGGGATTGTCCTGATAAAGGGCAGAATGGATCCGGTGCTGCTAAAGGGCCTG
TTCACAACTGTAAGTTTTTTTGTTTTTTGTTTTCCAGAAGAAACTGAATGGATTTTCTAGTTCTGTA
GTTTTGGTTTTGATATTAAGTGCTGCAATTTTTTGTTTTGTTTGGCATGTTTGTTTCATTTGTTTGT
TTCTCTAAATTAACATATGGTTGATACTAGTTCTGTTATTCCATGCTATCTATAGGCTATAGTTTTG
ATCCAATCTACAAATATAGTCATTAATTTCTGATTGTAATGTCTATTGTCTAACTTCCGGTAGTGGA
GATGTACTGTTTTTGGTCCTTTGTTACCTTAGGAAAAATTAAAACAGCTGAACATATGGTTATCTGT

ATGTTACAAAGTTACACGCGAGTCGTATCTCAATTCTATAGAAAACTGAATCCAGTTGATACAAATA
TTTGTGTGTATCTATTTTGACAATGAATCTCCACTTGAATCCTGATGCCTCATGATGAATTCAAATT
CACTCTGATGAATCAGATCCTGAACTTAGTGTAGGCAGCTGCTTTTTTGTCAACTTTGTATAGCTAA
CCACTTCTTTGTGTCATTTAATTTTTTATTTTAATTGAATCTTCAATTATTTTGAGGTTCGTATATG
ATGTATTAGCTTATAGTTTCACTATGTTACTTGTTTTTACTTAAAAATATAAGTTTTATTGATTATG
TGTCTTGCGATTCATAATAAGATTTGATTCACGATTTAGAAAATAGGTATTTCGATTCAGGATTTGA
GTCTTGATTTAATAATCATGCAGCTCCAAAAGTAGTGATAGTTTTCCTCTGAAGTCCTTTATTTTGA
CTCAAAATGAATTTTATGAGTCATAGGTGGCCAGTCTAGGTGGATACGGCCTATGGGTTCTTCATAT
TATAGAAGTTTGAGATTAGCATGTTTGGTTGTGGTTTGGTATACTGATAGTTTATTCTTGATTACAT
GTCAGAATTGCTAAAATCATTTTACCCTGTTAATATAGGGTGTTAAGAGTAAATTTTGTTGGTTGTT
ATATGCTTTTAATTTGATAATAATGAGAACATGAACAATCCAACCTTCTTGTTTCAGTTCTTGTTTG
AGATTTCACTCATAAACAGATGTTAAAACAAATGCGAGTGATAAAACGACTGTTAAATGCTTGGACC
CATGCCAAATGCGAACTGAAGTATTTTCCTAGTCGCAAATGTTAATATTAGAAATCAAATATTTAGA
GTGAAGTGTGTAAGTTTAGTAAGTTTAATGTTAACATGTTGCAAGTTATATGCAGCCCTTCCAGCCT
CTGCCTGAGCTCTTTCTGTATATCACCAATTAAATTGCATTCTATTGGATTGCTAAGCCTTTATAGT
CATCTGGTAGAAGGGTGCTTATATATATTATGTCTAATATGTTATTAACTCAATTTTTTCTTTCAGT
ACTGCGAACCAATGAGAGGCCCATAGGCCAGGTTACGAAGTTTGTGAGCGGAGACGATATGGAGGAT
GACTTCACGGCAGCACAAATAAAAGACGACAAGAGCAAACCTTCAAAGTTGAAAGATGACAATGTAA
AACCAAAGAAAGGTCCTAAAGTCGTGAACTTTGACTAAGTAGTTACTTCTTAGTGTTGTGTGGATAT
TTGTTTCCACTCTTCATTTTATTTGTTCTTCTAGTACCTCTAAGCCTACACTACACTCCTGCTATTC
ATTGGAATCTGCTGAATCTCATCTAGATTGAAATCAAATGCAACTTCTTCTGCAAACCCTGGGTTGT
CTGAAA

Gene *Ca04468*. *Cicer arietinum*, Desi, accession ICC-4958. Chromosome Ca4

>Ca_desi_V3.0_ref
GCACCACGAGATATTGAGGCATGATATGACTTAAATAAACGCAAAAAAATGAATTTTTAACAAATTT
TGAATCAAAATTATGTTTGTGGATTAAAACTAGGGAGAAATAGAATAGGGGAACTATTTTCGCGATT
CAGCTAAAATAAAGGGATCAAAACTGCAATTAAGCCTATATTTTATTAATATCAATTTTAAGAGATG
AAGAACAAATTGAACATATGCATGATACAGAGGCACATGGGTTGAGAATGAGAGAGACGAAATGCAA
TTTCAATTTCTCAAAATATAAACATAAAGGTGTGAGTATATTTTAATAATTTATCACTATTGTTTTG
ATANNNNNNNNNNNNNNNNNNNNNNNNNNNNNNNNNNNNNNNNNNNNNNNNNNNNNNNNNNNNNNNNNNN
NNNNNNNNNNNNNNNNNNNNNNNNNNNNNNNNNNNNNNNTAAAAGACACCGTAATTTAGGGTTGTGCAAA
AAATCCGATTATGAGACCCAAATTCAAAATGAGATCCAAATCCATTTTAAATATCCGATTAAAATCA
TATGGTTATAATCTGGTTGGATAACCACTTATNNNNNNNNNNNNNNNNNNNNNNNNNNNNNNNNNNNNNN
NNNNNNNNNNNNNNNNNNNNNNNNNNNNNNNNNNNNNNNNNNNNNNNNNNNNNNNNNNNNNNNNNNNNAA
ATAATAAATTTAATTATAAATCTAAATTCATATATTAATTTTAAAATAGGTATTGTTTGGTTTTTTT
TTAAAATCAAACATAAACATATAGCCTCTACCCCCTCCTGAAAAGGTCCGCGCTGTATACATTTTTT
AACCTAAATATTGATAGAAGTTACCTGCAAATATTTTATGACTTAATTATAAAAACAATCTAAAGAA
TTAAATGTATTTTGCCTAAAATAAATAAGAAATAAAAAATAAAAAAAAGGGGCAAAAGAAAGAAGAA
AACCCTGAGGTTTTAGATTGGGGTTATCTCGTCACCGCACCAGCTGCCGCAGCCGTATCTAACCTAA
TTAATCAATCAATCATCGGCCACATCTTGTCAGAGATGGTGAGCCAAAGGCAACGACTTGCTCGGAA
ACGATTCAAAGAAGAACACCCCGAACTATTCCCCAAAGTTGAACCCACTCCTCCTAAAGACCCACTC
AAAAAGAAGACCAAGAACAAATTCAAGCGCAAAAAGCCAGACTCAAAAGACAAACCCCGTTCAGGAA
AACGCCCCCTTCGTGTTCCCGGTATGAAACCCGGTGACACGTGTTTCATTTGCAAGGGCATAGATCA
TATCGCAAAATTCTGTACTCAAAAAGCCGAATGGGAAAAGAATAAGATATGTTTGCGGTGTCGACGA

CGCGGTCATAGAGCTCAGAATTGTCCTGAGGTTCATGATGGTTCTAAGGATGTTAAGTATTGTTATA
ATTGTGGTGATACTGGTCATTCTCTTGCTAATTGTCATCAACCTCTTCAAGAAGGAGGGACAATGTT
TGCTCAGTGTTTTGTTTGTAATCAACAAGGGCATTTGAGTAAAAATTGTCCTCAAAATGCTCATGGC
ATTTATCCTAAGGTATATTATCTTATATCATTTCAATTATACTATACAATTTTCTCTCCAAAGGATA
ACCATATAAGTTAGTTCAATTAACAACACTTGTTCTTTAAACAAACGGTTTGGCTCAAGTGATTAAT
GAGCTCTAGAGAATGACGGAGTGATCGGGATTATCACATTCGACCTCTAGTTGAAACAATTGTTGAC
GAGACTTTACTTATCTTCCGAATGAACTTTTAGATTATCAGTGTCCATTTCCCTTGAAAATCCGAGT
GTTAGAGAAAAAGAACAATAGTTGTTATTTGCAAGTAAGCTGTGTCTATATGCATACCTATTTATTT
ATTTGTTTTGTGTGTTTGTTTGTAATAGGGTGGTTGTTGTAAAATCTGTGGTGGTGTGACACATCTG
GCAAGGGATTGTCCTGATAAAGGGCAGAATGGATCCGGTGCTGCTAAAGGGCCTGTTCACAACTGTA
AGTTTTTTTGTTTTTTGTTTTCCAGAAGAAACTGAATGGATTTTCTAGTTCTGTAGTTTTGGTTTTG
ATATTAAGTGCTGCAATTTTTTGTTTTGTTTGGCATGTTTGTTTCATTTGTTTGTTTCTCTAAATTA
ACATATGGTTGATACTAGTTCTGTTATTCCATGCTATCTATAGGCTATAGTTTTGATCCAATCTACA
AATATAGTCATTAATTTCTGATTGTAATGTCTATTGTCTAACTTCCGGTAGTGGAGATGTACTGTTT
TTGGTCCTTTGTTACCTTAGGAAAAATTAAAACAGCTGAACATATGGTTATCTGTATGTTACAAAGT
TACACGCGAGTCGTATCTCAATTCTATAGAAAACTGAATCCAGTTGATACAAATATTTGTGTGTATC
TATTTTGACAATGAATCTCCACTTGAATCCTGATGCCTCATGATGAATTCAAATTCACTCTGATGAA
TCAGATCCTGAACTTAGTGTAGGCAGCTGCTTTTTTGTCAACTTTGTATAGCTAACCACTTCTTTGT
GTCATTTAATTTTTTATTTTAATTGAATCTTCAATTATTTTGAGGTTCGTATATGATGTATTAGCTT
ATAGTTTCACTATGTTACTTGTTTTTACTTAAAAATATAAGTTTTATTGATTATGTGTCTTGCGATT
CATAATAAGATTTGATTCACGATTTAGAAAATAGGTATTTCGATTCAGGATTTGAGTCTTGATTTAA
TAATCATGCAGCTCCAAAAGTAGTGATAGTTTTCCTCTGAAGTCCTTTATTTTGACTCAAAATGAAT
TTTATGAGTCATAGGTGGCCAGTCTAGGTGGATACGGCCTATGGGTTCTTCATATTATAGAAGTTTG
AGATTAGCATGTTTGGTTGTGGTTTGGTATACTGATAGTTTATTCTTGATTACATGTCAGAATTGCT
AAAATCATTTTACCCTGTTAATATAGGGTGTTAAGAGTAAATTTTGTTGGTTGTTATATGCTTTTAA
TTTGATAATAATGAGAACATGAACAATCCAACCTTCTTGTTTCAGTTCTTGTTTGAGATTTCACTCA
TAAACAGATGTTAAAACAAATGCGAGTGATAAAACGACTGTTAAATGCTTGGACCCATGCCAAATGC
GAACTGAAGTATTTTCCTAGTCGCAAATGTTAATATTAGAAATCAAATATTTAGAGTGAAGTGTGTA
AGTTTAGTAAGTTTAATGTTAACATGTTGCAAGTTATATGCAGCCCTTCCAGCCTCTGCCTGAGCTC
TTTCTGTATATCACCAATTAAATTGCATTCTATTGGATTGCTAAGCCTTTATAGTCATCTGGTAGAA
GGGTGCTTATATATATTATGTCTAATATGTTATTAACTCAATTTTTTCTTTCAGTACTGCGAACCAA
TGAGAGGCCCATAGGCCAGGTTACGAAGTTTGTGAGCGGAGACGATATGGAGGATGACTTCACGGCA
GCACAAATAAAAGACGACAAGAGCAAACCTTCAAAGTTGAAAGATGACAATGTAAAACCAAAGAAAG
GTCCTAAAGTCGTGAACTTTGACTAAGTAGTTACTTCTTAGTGTTGTGTGGATATTTGTTTCCACTC
TTCATTTTATTTGTTCTTCTAGTACCTCTAAGCCTACACTACACTCCTGCTATTCATTGGAATCTGC
TGAATCTCATCTAGATTGAAATCAAATGCAACTTCTTCTGCAAACCCTGGGTTGTCTGAAA

<u>Gene *Ca07571*</u>. *Cicer arietinum*, Kabuli, cv. Frontier. Chromosome Ca5, Reverse-Compliment

>NC_021164. ASM33114v1, whole genome shotgun sequence

ACTACCCACACCCCTCAAATGTTTAAAAAAATTAATATTCCCAAATTACCCTCTCCTTCTTCCTCTT
CATTAACCTAACTCTTCTTCTTCTTCTTCTTCTTCTTTATTCTATCTTCATCATCTTCATTCAACAC
ATTCTTCATCTTCTTCATTTTATTCAACCTTAACCATCATTATCATTAACCATCTTCATAAATCATC
ATCAATCATCATTAATTATCATCAATCACCATCAATCCCCATCAATCATCATTAATTCAATATTTTG
CTAACATTTACGTGAATTCAATTTCACGTAAGTTTACGTGAACTAAGTTCACGTAGGTTTACGTAAA
CTGAATTCTAGAAAAGATACGTGAACTCAGTTCACGTACACCTACGTGAATTCACTTCACGTAAACT
TACGTGAAATTGAGTTCACGTATGTTTGATTTTCAATCTTCAATTATTTCAAAATCTTAATTCACAC
ATACGTGAATTAAGATTGCGTATAGACTGAGATGTTTACGCAATTTCTCAGTTCACGTACATGGCTT
ATTATTCAACATTCAATGATTACGCAATCTTAGTTCACGTAGGTGTACGTGAATTCAGATTGCGTAC
ACTATGAGGATTCAAGTTTCAAACTCCATTGATTATTGCAATATGAGTTTACGTATATGTACGTGAAT
TCAAATTGCGTACAGTCTGAGATTTTTAATTTTTTTTAAACATACGTGAACTAAGTTCACGTAACTT
CAGAACGTACGTGAACTGAGTTCACGTAAAATACCCAGATTTAAAAATAAAATAAAATAAAACACAC
CTTTTTGTTGGTGAGGTATGATGATTTTTGTATGAGTTATGAG**G**TGTGATGAAAGTTGATAAATGTG
AGTTATGAGATAATAAATGTGTAACATTGATGGCAGGGGCATTTTGGGTATTTTGATTTTTTTTAAA
TTTTGAGGGGTGTGAATAGCAATTTGGGGGGTGTTGGTAGCAATATTGTTATTATTATTATTTTTAA
AACTGAAAAACACAAAAAAGAAAAAAACTTAGAATATCTGAGAGGTGTGTGTATGATTAGGGCTTGC
AAGTGTGAACTCTTTGCGGCATAGGAAGGCGAATCGATGATGGCTAGAGATCACGAACAAGTCGCCG
AAATTAACGACAATCTCGATGGCGCTTCAACTCCATCGCTCGTTTTCAGCAGCGACGATGACGACGA
GGAAGCGAATCAAGATCTGAGCCTCAAGATCGTGGAAAAAGCCATGCGAACGCGAGAGGCCAAACTC
TCCCCAAACGACGACGTATCGGATGAACCCAGCGGTCCCGCTGCTTTCGAAGTAATGGAAGAGTCCC
TGAATCGAAGTGTATGTCTTATTCCGTGTTTGGTTTCTTTTATTTATTTTTAAATGATTTTGTTGAT
TTTGTTAATTCGAACTTCCTTAGTGTTTAAATCAATTTTGATAGATTGAATTAAACACGAAAACTAT
CCCTTTAGGCATCAACCAGGTTATGATTACATTGAAAATTGAAATTGTTTTTTAAGGGGAACAAAGT
TGAAACTGGAACACTGAAATGTTTATTAAAAAATTGCATTGTAACATGTAGTTTGCAAAGCTTATAA
CTTAAATGTTTGGGGAATTTGTCGCTGAGAATCTAATTCATGAATAGACAATCACATTTTCAGTAGT
ATATTTTTGATTTTGTTAATGTTATTCTTTTCTATGTAGGTTATTATAGCTGCAGAACAAGAGGTGG
AAGAGATAATCAAAACCACTGAGAAGGATGAATCTGTGGAAGCAAGTGCTGTTCAGATAGGTGACAA
CGCTGTTCTGCGAAAGCTGCTTGTGAGTTGATATATTTGATCCACAATGTGATTGATTTTCAAAATA
CTGTACTCTGAGGAATGCTTAGTTGAGTATGTCACTCGATGTTTCTTTTCTATCAGCGGGGTCCAAG
GTATTTTGACCCACCAGATAGTAGTTGGGGAGCATGCTATAATTGTGGCGAGGAAGGTCATGCTGCT
GTAAACTGTACAGCAGCAAAGCGCATGAAACCATGCTATGTATGTGGTGGTTTGGGACACGGTGCTA
AGCAATGTACTAAGGTATGTCTACTTGCCTAACGGATTATGAAGGTTTAAAGTTTTTGTTTAGTATA
CTGATGAGATCTGCTTGATGCTCATGTAGGCCCAGAGTTGCTTTATCTGTAAGAAAGGTGGCCACCG
GGCCAAAGACTGTCCTGAGAAGCTCATGACTGCACGTGTTTCTAAAAGCCTGACAATTTGCTTAAAG
TGTGGAAATTCGGGGCATGATATGTTTTCATGCAAGAATGATTATTCACGGGATGATCTCAAGGTTG
TTATGTTGCTTGTTCTTTTTTTCTCCTATGTATCAAGTTTGCTAAAGGTTCTTGGTTGGGTTTACATT
AGAATATGGCTTACATTGGATGTGAAATTTTCAGTCAGTGATTCCTTATCTCTTTCTTCTGTTTAAT
TAGGAGATTCAATGTTATCTCTGCAAGACATTTGGACACTTGTGTTGTGTCAATACTGTTGATGCAA
TACCGGGAGAAATTTCTTGTTACAAATGTGGTCAGATGGGTCATACCGGTTTGGTAAGTTTTATTTA
ATGAATTTTATTTAATAACTTATGTGCCTTTCCAGAGATAAGCATCTCTATTTTGCATAATGTAGTC
TATGACTCAGTCTCTCTTTACAATGGAAATGTATGACTCGGTCTCTCTTTACATACTACTTAGTAAT
TACTAGATAGTTGAGACACTAAAATGTTCTCAAGATCAACATTTCATTTAATATATCTAAATTATAG
TATAATAAAAACTCAAAAAATATCATGAAAATATCTTCCAATCAGTACCCTTAAATTTCTTTGTGAC
ATGACATGTCAGTGTTGTTAATCACAGAAAATAGTGATTTGTTCAAATTTCGTTATGCTACAATACT

ATAGTGCCTCTATAGCCGTTATTTGACAACACTGTGACATGTATGGTAATGAATAACACTAAAAGTG
ACTTTGTGATGGAAACTTATTATGCCTATCATTACCTTTTGAACTTGCTTGATTCCCTATGAACTTT
TACTCACAATGCGTTAATAGTAGTTTAATCTCGTGGTATTGGCAACCAAAGAGGACTTCAGCTAAAG
CTTGATCTAACACATGAAATGCTTCCTTTTAAATAAAACTATTGGCAAGTATAAATGTGAGAATCAA
TTGATGCAGGCATGCTCAAGGTTGCAAAGCGAGACTACTGGTGCTGCTTCACCTAGTTTGTGCTATA
GGTGTGGTGAAGTAGGACATTTTGCCCGAGAATGCACAAGCTCAACCAAGGTACATTTAAAAAATTA
TATAAGGTTACTAAAATTAATACAGCCGTTTGCCTTTTCTTTTATTGTTTAATTTTCATGTTGACTC
GTGTAATAAAGTAAGTAATTTCTTGGCTGCTGTGTTTTCCTTCACAGGCTGGGAAAAAGAATAGTGA
ATTTTCAAATACAAAAAAAAGAAGATCCTATAAAGAAAATGATTTCAGGGGACATTGGTCTGCACCT
CATGATGCGGGTAAGATGCATAAAAAGAAGCGACCTCTTCCAGACGAAAGAGGCTTTACAACCCCCA
AGAAATCAAAGAGTAGAGGTGGCTGGTCGAGGGAGCTTCCCACCGAAGAAAGAGGCCTTACAACCCC
CACAAAATCAAGGAGTAGAGGTGGCTGGACAATGGAACATCCTGCAGAAGAAAGAAACTTTAAAAGA
GGCTTTACAACCCCCAAGAAATCAAAGAGTAGAGGTGGCTGGACAATGGAGCACCCTGCAGATGAAA
GAGACTTTAATTCTCCTAAAAAATTCAAGAGCAGAGGTGGCTGGACGGCTGAGTATGCCGGAGAATT
CTCTTCTTCAAAGTCCAAAAGGAGCAGTTGGAGGTCTCCAGGGACACCATCTGCTAGAAGCACTAAA
ATTCACTCGCGAAGTAGTGGAAGTCAAACTCCAGGTTGGAGTTATAAATCTTCTCAGGGGTGGCAGG
GTCAACAACCTGGAGCCTCAAATTACCAAGGATTAGCCATGGATTTCCATCATAGATATTCAGCATC
AAGGTTTGGCAACTCTAGCAGTGATGGATATAGGAGAAGTCATTGGCAGTAGCTTTCGCCTTCGTGT
TCTTTACAATGTCATCTGTAAACTACTTGTAAAATGACACCTAGTTGCATGTTTTTATTTCTTCAA
AATAATAATATTTATTTTCCCTTCAGTTATTCATTCAA

Gene *Ca07571. Cicer arietinum*, Desi, accession ICC-4958. Chromosome Ca5, Reverse-Compliment

>Ca_desi_V3.0_ref
TAATGTTATACAAATTAAATTATTATTATTATTACAATATTGCTACCAACACCCCCTAATTTACTAC
CCACACCCCTCAAATGTTTAAAAAAATTAATATTCCCAAATTACCCTCTCCTTCTTCCTCTTCATTA
ACCTAACTCTTCTTCTTCTTCTTCTTCTTCTTTATTCTATCTTCATCATCTTCATTCAACACATTCT
TCATCTTCTTCATTTTATTCAACCTTAACCATCATTATCATTAACCATCTTCATAAATCATCATCAA
TCATCATTAATTATCATCAATCACCATCAATCCCCATCAATCATCATTAATTCAATATTTTGCTAAC
ATTTACGTGAATTCAATTTCACGTAAGTTTACGTGAACTAAGTTCACGTAGGTTTACGTAAACTGAA
TTCTAGAAAAGATACGTGAACTCAGTTCACGTACACCTACGTGAATTCACTTCACGTAAACTTACGT
GAAATTGAGTTCACGTATGTTTGATTTTCAATCTTCAATTATTTCAAAATCTTAATTCACACATACG
TGAATTAAGATTGCGTATAGACTGAGATGTTTACGCAATTTCTCAGTTCACGTACATGGCTTATTAT
TCAACATTCAATGATTACGCAATCTTAGTTCACGTAGGTGTACGTGAATTCAGATTGCGTACACTAT
GAGGATTCAAGTTTCAAACTCCATTGATTA**C**GCAATATGAGTTTACGTATATGTACGTGAATTCAAA
TTGCGTACAGTCTGAGATTTTTAATTTTTTTAAACATACGTGAACTAAGTTCACGTAACTTCAGAA
CGTACGTGAACTGAGTTCACGTAAAATACCCAGATTTAAAAATAAAATAAAATAAAACACACCTTTT
TGTTGGTGAGGTATGATGATTTTTGTATGAGTTATGAG**A**TGTGATGAAAGTTGATAAATGTGAGTTA
TGAGATAATAAATGTGTAACATTGATGGCAGGGGCATTTTGGGTATTTTGATTTTTTTTAAATTTTG
AGGGGTGTGAATAGCAATTTGGGGGGTGTTGGTAGCAATATTGTTATTATTATTATTTTTAAAACTG
AAAAACACAAAAAAGAAAAAAACTTAGAATATCTGAGAGGTGTGTGTATGATTAGGGCTTGCAAGTG
TGAACTCTTTGCGGCATAGGAAGGCGAATCGATGATGGCTAGAGATCACGAACAAGTCGCCGAAATT
AACGACAATCTCGATGGCGCTTCAACTCCATCGCTCGTTTTCAGCAGCGACGATGACGACGAGGAAG
CGAATCAAGATCTGAGCCTCAAGATCGTGGAAAAAGCCATGCGAACGCGAGAGGCCAAACTCTCCCC
AAACGACGACGTATCGGATGAACCCAGCGGTCCCGCTGCTTTCGAAGTAATGGAAGAGTCCCTGAAT
CGAAGTGTATGTCTTATTCCGTGTTTGGTTTCTTTTATTTATTTTTAAATGATTTTGTTGATTTTGT
TAATTCGAACTTCCTTAGTGTTTAAATCAATTTTGATAGATTGAATTAAACACGAAAACTATCCCTT

TAGGCATCAACCAGGTTATGATTACATTGAAAATTGAAATTGTTTTTAAGGGGAACAAAGTTGAAA
CTGGAACACTGAAATGTTTATTAAAAAATTGCATTGTAACATGTAGTTTGCAAAGCTTATAACTTAA
ATGTTTGGGGAATTTGTCGCTGAGAATCTAATTCATGAATAGACAATCACATTTTCAGTAGTATATT
TTTGATTTTGTTAATGTTATTCTTTTCTATGTAGGTTATTATAGCTGCAGAACAAGAGGTGGAAGAG
ATAATCAAAACCACTGAGAAGGATGAATCTGTGGAAGCAAGTGCTGTTCAGATAGGTGACAACGCTG
TTCTGCGAAAGCTGCTTGTGAGTTGATATATTTGATCCACAATGTGATTGATTTTCAAAATACTGTA
CTCTGAGGAATGCTTAGTTGAGTATGTCACTCGATGTTTCTTTTCTATCAGCGGGGTCCAAGGTATT
TTGACCCACCAGATAGTAGTTGGGGAGCATGCTATAATTGTGGCGAGGAAGGTCATGCTGCTGTAAA
CTGTACAGCAGCAAAGCGCATGAAACCATGCTATGTATGTGGTGGTTTGGGACACGGTGCTAAGCAA
TGTACTAAGGTATGTCTACTTGCCTAACGGATTATGAAGGTTTAAAGTTTTTGTTTAGTATACTGAT
GAGATCTGCTTGATGCTCATGTAGGCCCAGAGTTGCTTTATCTGTAAGAAAGGTGGCCACCGGGCCA
AAGACTGTCCTGAGAAGCTCATGACTGCACGTGTTTCTAAAAGCCTGACAATTTGCTTAAAGTGTGG
AAATTCGGGGCATGATATGTTTTCATGCAAGAATGATTATTCACGGGATGATCTCAAGGTTGTTATG
TTGCTTGTTCTTTTTTCTCCTATGTATCAAGTTTGCTAAAGGTTCTTGGTTGGGTTTACATTAGAAT
ATGGCTTACATTGGATGTGAAATTTTCAGTCAGTGATTCCTTATCTCTTTCTTCTGTTTAATTAGGA
GATTCAATGTTATCTCTGCAAGACATTTGGACACTTGTGTTGTGTCAATACTGTTGATGCAATACCG
GGAGAAATTTCTTGTTACAAATGTGGTCAGATGGGTCATACCGGTTTGGTAAGTTTTATTTAATGAA
TTTTATTTAATAACTTATGTGCCTTTCCAGAGATAAGCATCTCTATTTTGCATAATGTAGTCTATGA
CTCAGTCTCTCTTTACAATGGAAATGTATGACTCGGTCTCTCTTTACATACTACTTAGTAATTACTA
GATAGTTGAGACACTAAAATGTTCTCAAGATCAACATTTCATTTAATATATCTAAATTATAGTATAA
TAAAAACTCAAAAAATATCATGAAAATATCTTCCAATCAGTACCCTTAAATTTCTTTGTGACATGAC
ATGTCAGTGTTGTTAATCACAGAAAATAGTGATTTGTTCAAATTTCGTTATGCTACAATACTATAGT
GCCTCTATAGCCGTTATTTGACAACACTGTGACATGTATGGTAATGAATAACACTAAAAGTGACTTT
GTGATGGAAACTTATTATGCCTATCATTACCTTTTGAACTTGCTTGATTCCCTATGAACTTTTACTC
ACAATGCGTTAATAGTAGTTTAATCTCGTGGTATTGGCAACCAAAGAGGACTTCAGCTAAAGCTTGA
TCTAACACATGAAATGCTTCCTTTTAAATAAAACTATTGGCAAGTATAAATGTGAGAATCAATTGAT
GCAGGCATGCTCAAGGTTGCAAAGCGAGACTACTGGTGCTGCTTCACCTAGTTTGTGCTATAGGTGT
GGTGAAGTAGGACATTTTGCCCGAGAATGCACAAGCTCAACCAAGGTACATTTAAAAAATTATATAA
GGTTACTAAAATTAATACAGCCGTTTGCCTTTTCTTTTATTGTTTAATTTTCATGTTGACTCGTGTA
ATAAAGTAAGTAATTTCTTGGCTGCTGTGTTTTCCTTCACAGGCTGGGAAAAAGAATAGTGAATTTT
CAAATACAAAAAAAAGAAGATCCTATAAAGAAAATGATTTCAGGGGACATTGGTCTGCACCTCATGA
TGCGGGTAAGATGCATAAAAAGAAGCGACCTCTTCCAGACGAAAGAGGCTTTACAACCCCCAAGAAA
TCAAAGAGTAGAGGTGGCTGGTCGAGGGAGCTTCCCACCGAAGAAAGAGGCCTTACAACCCCCACAA
AATCAAGGAGTAGAGGTGGCTGGACAATGGAACATCCTGCAGAAGAAAGAAACTTTAAAAGAGGCTT
TACAACCCCCAAGAAATCAAAGAGTAGAGGTGGCTGGACAATGGAGCACCCTGCAGATGAAAGAGAC
TTTAATTCTCCTAAAAAATTCAAGAGCAGAGGTGGCTGGACGGCTGAGTATGCCGGAGAATTCTCTT
CTTCAAAGTCCAAAAGGAGCAGTTGGAGGTCTCCAGGGACACCATCTGCTAGAAGCACTAAAATTCA
CTCGCGAAGTAGTGGAAGTCAAACTCCAGGTTGGAGTTATAAATCTTCTCAGGGGTGGCAGGGTCAA
CAACCTGGAGCCTCAAATTACCAAGGATTAGCCATGGATTTCCATCATAGATATTCAGCATCAAGGT
TTGGCAACTCTAGCAGTGATGGATATAGGAGAAGTCATTGGCAGTAGCTTTCGCCTTCGTGTTCTTT
ACAATGTCATCTGTAAACTACTTGTAAAATGACACCTAGTTGCATGTTTTTTATTTCTTCAAAATAA
TAATATTTATTTTCCCTTCAGTTATTCATTCAA

Supplementary material S4. Results of SNP genotyping in *Ca04468* and *Ca07571* genes using ASQ method.

Genotyping of 12 F_6 breeding lines, 4 plants in each line, compared to parents Krasnokutsky-123 and ICC-12654 for *Ca04468*-SNP1 using ASQ method in 96-well microplate. Red and blue colour indicated for fluorescence registration, FAM or HEX, respectively.

Plate 364. Chickpea-Ca04468. ♀Krasnokutsky-123 × ♂ICC-12654. 06.12.2022.												
	1	2	3	4	5	6	7	8	9	10	11	12
A	Kras-nok-1	H18-1-1	H18-3-1	H18-5-1	Kras-nok-5	H18-7-1	H18-9-1	H18-11-1	Kras-nok-9			
B	Kras-nok-2	H18-1-2	H18-3-2	H18-5-2	Kras-nok-6	H18-7-2	H18-9-2	H18-11-2	Kras-nok-10			
C	Kras-nok-3	H18-1-3	H18-3-3	H18-5-3	Kras-nok-7	H18-7-3	H18-9-3	H18-11-3	Kras-nok-11			
D	Kras-nok-4	H18-1-4	H18-3-4	H18-5-4	Kras-nok-8	H18-7-4	H18-9-4	H18-11-4	12654-9			
E	12654-1	H18-2-1	H18-4-1	H18-6-1	12654-5	H18-8-1	H18-10-1	H18-12-1	12654-10			
F	12654-2	H18-2-2	H18-4-2	H18-6-2	12654-6	H18-8-2	H18-10-2	H18-12-2	12654-11			
G	12654-3	H18-2-3	H18-4-3	H18-6-3	12654-7	H18-8-3	H18-10-3	H18-12-3	NTC			
H	12654-4	H18-2-4	H18-4-4	H18-6-4	12654-8	H18-8-4	H18-10-4	H18-12-4	NTC			

Genotyping of 12 F_6 breeding lines, 4 plants in each line, compared to parents ICC-10945 and Louch for *Ca07571*-SNP4 using ASQ method in 96-well microplate. Red and blue colour indicated for fluorescence registration, FAM or HEX, respectively.

Plate 401 Chickpea-Ca07571. ♀ICC-10945 × ♂Looch. 01.03.2023.												
	1	2	3	4	5	6	7	8	9	10	11	12
A	10945-1	H35-1-1	H35-3-1	H35-5-1	10945-5	H35-7-1	H35-9-1	H35-11-1	10945-9			
B	10945-2	H35-1-2	H35-3-2	H35-5-2	10945-6	H35-7-2	H35-9-2	H35-11-2	10945-10			
C	10945-3	H35-1-3	H35-3-3	H35-5-3	10945-7	H35-7-3	H35-9-3	H35-11-3	10945-11			
D	10945-4	H35-1-4	H35-3-4	H35-5-4	10945-8	H35-7-4	H35-9-4	H35-11-4	Louch-9			
E	Louch-1	H35-2-1	H35-4-1	H35-6-1	Louch-5	H35-8-1	H35-10-1	H35-12-1	Louch-10			
F	Louch-2	H35-2-2	H35-4-2	H35-6-2	Louch-6	H35-8-2	H35-10-2	H35-12-2	Louch-11			
G	Louch-3	H35-2-3	H35-4-3	H35-6-3	Louch-7	H35-8-3	H35-10-3	H35-12-3	NTC			
H	Louch-4	H35-2-4	H35-4-4	H35-6-4	Louch-8	H35-8-4	H35-10-4	H35-12-4	NTC			

Supplementary material S5. Sequences and genetic positions of the used DArT markers for analysis of *Ca04468* and *Ca07571* genes.

DArT clone	Position on chromosome	Sequence
Chromosome Ca4: Genetic region around *Ca04468* gene		
13146196	11,925,658	CAACATCTCCATCTATGCCAAGAAGTCCAGCTAGAGCAGATAGTCCAATGAGCTCTGGTTCACG
50759538	12,249,346	AATTAGAGTGAGAAAACAGCGAGGAATTGGTAACAGTTACAGATCGGAAGAGCGGTTCAGCAGG
5824740	12,548,932	TAATACATTCGCATGCTTCATAATGAAGACATTTATACATATTGACATGCTTCATAATATTTCA
5824874	12,575,279	TGGATGGTGTATCTATCATACATCATAGTACATAGACCATCTAAGATTAGATCTGTAGTCCAGG
5826066	13,746,542	AATCAGAAAGTTTATTCTTGGACTGTGAAAATGAATTACAGATCGGAAGAGCGGTTCAGCAGGA
5825634	13,747,318	AATCAGAAAGTTTATTCTTGGACTGTGAAAATGAAATAAGGCAAGAATTGAGATGGCTCAAAGC
10263417	14,030,024	AAACCAAAGGATCGGTGTAAAAATAAAATTATTTTATTTTTCTTCCTTTTGAATTATATTACAG
10263426	14,031,447	CAGACCATTTTGGTATATATATCATCTTACTTATTTCTCTTATGTTTCAAAATAAGAGAATTAC
Chromosome Ca5: Genetic region around *Ca07571* gene		
10269623	39,519,469	CATGTACTGTTATGGATAGGGGTGTGTTCCTCACGCCAACCATATTCTTCCCCGTCCTTCATTG
23888542	39,867,964	GTAGTCGACTTCCGAAGAGTGTTTGCAGCAACGTGTTACAGATCGGAAGAGCGGTTCAGCAGGA
23888044	40,194,472	GGTGAATGATCCGGTGGCTATGAAACTACTTGGCAAGGCAGGAGAGATGGCCTCTCTGGAGGCT
23889105	40,214,345	GCAAACCTGTTCCTGAATTCGAATACACTCCACGCAGCGTCTCTCACTTGCACCATCTTTTACA
23886128	40,542,530	GTGAAAATATAGTGCAGAACAGAAAAATAATTGAGAACAATTTCAGAATATTGTAAGCCATGAT
10258584	40,673,626	TACAGTCTGGTATCATTTGACCTATGTTTCGTATGGAATTATCCACTTATTTACCAAAATTACC
23888403	41,121,028	AGTTTGAGCGGACCTGTCTCAGCTACCCTTTACCTTGGCTATTCATTACAGATCGGAAGAGCGG
35486562	41,395,271	CAGTCAAATTTCCAGCATTTGGCCCATGTAACCAGTCAAGTATAAAAGTACACAGGCACTCTGC
